# Quantifying milk yield-dependent aflatoxin B1-to-M1 transfer in dairy cows: a Bayesian consensus toxicokinetic model

**DOI:** 10.1007/s00204-026-04366-3

**Published:** 2026-04-28

**Authors:** Jan-Louis Moenning, Jorge Numata

**Affiliations:** Department Food and Feed Safety in the Food Chain, German Federal Institute for Risk Assessment (BfR), Max-Dohrn-Strasse 8-10, 10589 Berlin, Germany

**Keywords:** Pharmacokinetics, Hierarchical Bayesian inference, Mycotoxins, Milk, Feed risk assessment, Feed risk management

## Abstract

Aflatoxin B1 (AFB1) is a hepatocarcinogenic mycotoxin found in animal feed contaminated by mold from the genus Aspergillus. It can be transferred into food of animal origin, such as the milk of dairy cows, as the metabolite aflatoxin M1 (AFM1), which has 10% of the potency of AFB1. A multitude of studies has been conducted over the last decades to quantify the transfer of AFB1 as AFM1 in milk. The transfer rates derived from these studies vary considerably, with one variability source being the different milk yields of the experimental animals. A simple deterministic model was developed in the past to predict the influence of milk yield on the transfer rate in a steady-state setting. Although that previous model could predict transfer, it lacked dynamic predictions and did not quantify uncertainty, as is necessary for risk analysis. In this work, we synthesized transfer data from 19 published controlled dairy cow feeding studies (AFB1 doses: 6.6–2800 µg/d up to a 20,000 µg single dose ; milk yields: 6.4–46.4 L/d) using a hierarchical Bayesian approach with a reparametrized two-compartment toxicokinetic model designed to overcome parameter correlations, capture between-study heterogeneity and quantify prediction uncertainty. The model communicates prediction uncertainty using milk yield-dependent credible intervals (CI) for concentration profiles in milk across studies. It predicts a median transfer rate of 0.41% (95% CI: 0.13–1.40%) at 5 L/d, increasing to a median of 3.05% (95% CI: 1.07–8.19%) at 50 L/d. The model can be used in risk assessment and risk management to quantitatively predict transfer including credible intervals.

**Supplementary Information:**

The online version contains supplementary material available at 10.1007/s00204-026-04366-3.

## Introduction

Aflatoxins are mycotoxins produced by three mold species of the genus Aspergillus (*Aspergillus. flavus*, *A. parasiticus* and *A. minisclerotigenes*) in some crops, especially in humid and warm climates (Schamann et al. 2024). Aflatoxins display a number of different toxic effects, such as cytotoxicity and oxidative stress induction (Dohnal et al. 2014; Benkerroum 2020). The International Agency for Research on Cancer (IARC) classified aflatoxin B1 as Group 1 and M1 as Group 2B human carcinogens (IARC 2012). The WHO Global Burden of Disease report emphasizes aflatoxins as a significant foodborne health risk, with even regular low-level consumption causing serious health effects (WHO 2015). Aflatoxin B1 (AFB1) is the most common aflatoxin found in food and feed of vegetable origin and displays the highest toxic potential among aflatoxins (Kondampati and Srinu 2023). Aflatoxins can also be found in feed for dairy cows (Streit et al. 2013). AFB1 is not directly transferred in relevant quantities from feed in milk. However, AFB1 is metabolized in the cow’s liver into aflatoxin M1 (AFM1), among other metabolites. It has been shown that, unlike the other metabolites, AFM1 is transferred in significant quantities in milk (EFSA 2020). While AFM1 is considered to have 10% of the toxic potency of AFB1 (EFSA 2020), it still exhibits a significant toxic effect.

The EU has set maximum levels (MLs) for AFB1 of 20 µg/kg 88% DM in feed material and 5 µg/kg 88% DM in compound feed for dairy cattle (EU Regulation 574/2011). Furthermore, MLs of 0.050 µg/kg of AFM1 in milk and milk destined for dairy products, as well as 0.025 µg/kg of AFM1 for infant formula and follow-on milk, are set by (EU Regulation 915/2023). While several studies have been conducted describing the transfer of AFB1/M1, the resulting transfer rates (TRs) differ drastically between studies (Zentai et al. 2023). One potential explanation for this discrepancy is the variability in milk yields of the experimental animals, resulting in different excretion rates of AFM1. Therefore, past efforts have been made to develop models that describe this transfer and take milk yield into account. One such toxicokinetic (TK) model was developed by van Eijkeren (Van Eijkerenr et al. 2006), which describes the milk yield-dependent transfer. However, it has three limitations: 1) it only provides an estimate of the steady state transfer; 2) it is parameterized on a very limited dataset; and 3) it does not provide an estimate of its prediction uncertainty. This work aims to address these limitations by extending the model to a dynamic setting and using a large number of datasets to derive appropriate credible intervals for communicating uncertainty. To achieve this, we synthesized data from several published studies covering a wide range of milk yields and dosages in order to parameterize the consensus model. The parameterization is performed using a hierarchical Bayesian inference approach. A potential distribution of model parameters is derived for each study, which are linked via overarching parameters describing the distribution of these study-specific parameters across all studies synthesized. The resulting model communicates prediction uncertainty, allowing for more informed decision-making regarding the risk analysis of AFB1 contamination in feed. The model is made available as computer code in the supplements and on the web as an interactive calculation in the graphical web tool ConTrans.bfr.bund.de.

## Materials and methods

### Data acquisition

To parameterize the model, relevant studies on aflatoxin B1/M1 transfer to cow’s milk with sufficient data and quality were identified in the literature. Only controlled feeding studies that clearly report the milk yield and the amount of AFB1 in the feed per day were included. From each study, we used the groups where the only treatment was exposure to aflatoxin B1 (e.g., no use of mycotoxin binders). The studies can be broadly divided into three categories: full concentration–time profiles (ct-profiles, n = 6), where measurements were performed in the assimilation and depuration phases; depuration phase ct-profiles (n = 3), where the assimilation phase had no measurements; and transfer rate (TR) reported only (n = 10). For including studies with ct-profiles in milk or blood, we require that the exact feeding and milking times were reported. For including studies in which we only used the reported TR, we required the study to specify that the TR had been calculated at steady state. A special case was the publication by Guo et al. (Guo et al. 2021), where a single dose study was performed in addition to the continuous dosing study, and blood concentrations of AFB1 and AFM1 were also reported. Therefore, the results of this study were treated as two different studies. For 3 publications, it was possible to obtain the raw data (Walte et al. 2022) (Guo et al. 2021) and (Lamp et al. 2026); for the rest of the publications, the data was scraped from the publications. Milk yields in the studies ranged from 6.4 L/d–46.4 L/d and doses ranged from 6.6–2800 µg/d up to a single dose of 20000 μg. Further details of the data used from these 19 studies are provided in Table 1 and in the Supplement.

**Table 1:** Dose and average milk yield of studies used for deriving the consensus model. If a study had animals with vastly different milk yields, they are shown separately

Reference	Dose $[\mu g /d]$	Average milk yield [L/d]	Breed	Available data
Walte et al. (2022)	50	32.5	German Holstein, black and white	Full milk ct-profile
Lamp et al. (2026)	91.2	34	German Holstein, black and white	Full milk ct-profile
Guo et al. (2021)	2000	10.9	Holstein	Full milk ct-profile
Guo et al. (2021)	20,000 (single dose)	10.9	Holstein	Full milk + blood ct-profile
Britzi et al. (2013)	80	44.7, 29.8	Israeli-Holstein	Full ct-profile
Jiang et al. (2018)	1725	38.4	Holstein	Full ct-profile
Masoero et al. (2007)	98.1	21.5, 43, 21.7, 34.5	Holstein	Full ct-profile
Queiroz et al. (2012)	1725	18.9	Holstein	Depuration milk ct-profile
Rodrigues et al. (2019)	2800	36.3	Holstein	Depuration milk ct-profile
Xiong et al. (2015)	340, 696	21.3, 22.4	Holstein	Depuration milk ct-profile
Kutz et al. (2009)	2500	34.19	Holstein	TR in milk only
Pietri et al. (2009)	97.3	31.2	Italian Friesien	TR in milk only
Giovati et al. (2014)	102	32.1	Holstein Friesian	TR in milk only
Rojo et al. (2014)	880	27.9	Holstein	TR in milk only
Maki et al. (2016)	2340	21.3	crossbred (Holstein × Jersey × Norwegian Red)	TR in milk only
Galvano et al. (1996)	60	18.5	Friesian	TR in milk only
Frobish et al. (1986)	349—2491	14.6—32.3	Holstein	TR in milk only
Price et al. (1985)	6.6—740	27.7	Holstein	TR in milk only
Chopra et al. (1999)	71—342	7.4	crossbred	TR in milk only
Veldman et al. (1992)	28.6–58	6.4–46.4	NA	TR in milk only

### Model structure

**Fig. 1:**
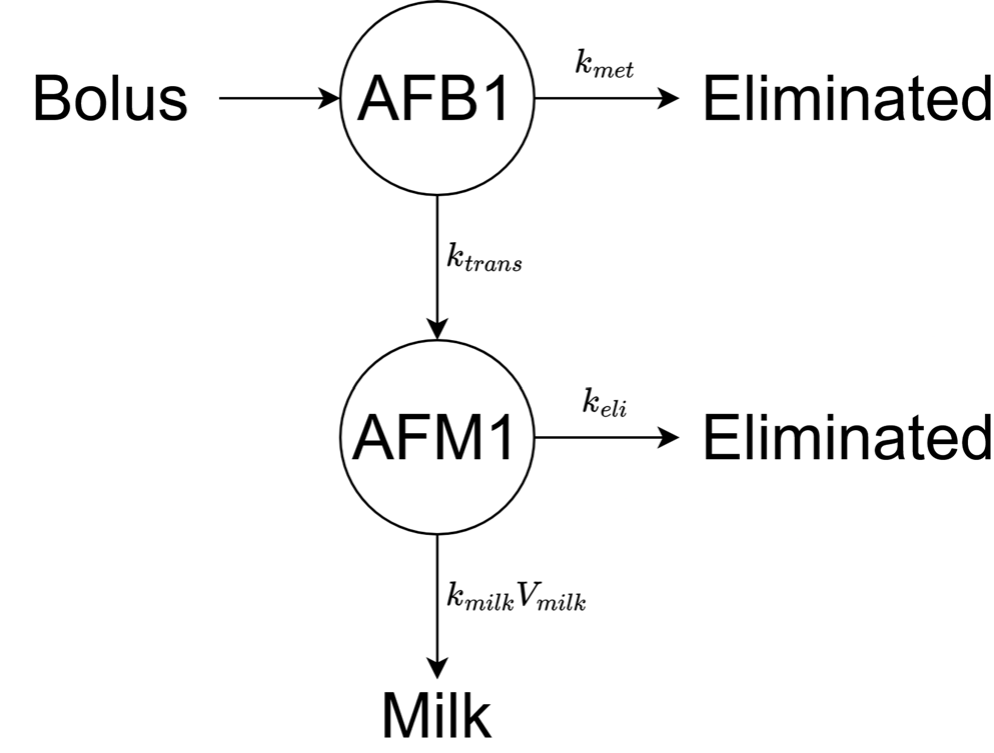
Schematic description of the aflatoxin model, describing the transformation of AFB1 into AFM1 and its subsequent elimination

The model developed here is an extension of the toxicokinetic model by (van Eijkeren et al. 2006). It consists of two compartments representing the aflatoxin B1 (AFB1) and aflatoxin M1 (AFM1) amounts in the cow (Fig. 1). AFB1 can be directly eliminated (${k}_{met}$) without being transformed into AFM1 either by being metabolically transformed into a different metabolite than AFM1, not being absorbed in the gastrointestinal tract and/or being excreted via a non-milk pathway e.g., urine. The substance can transition from the AFB1 compartment to the AFM1 compartment via biotransformation (${k}_{trans}$) but not in reverse. AFM1 is then eliminated either via excretion with milk (${k}_{milk}{V}_{milk}$), which is assumed to be proportional to milk yield (${V}_{milk}$), or through non-milk pathways (${k}_{eli}$), such as the excretion of AFM1 or its further metabolites with urine. The small molar mass difference between AFB1 and AFM1 is ignored because it is indirectly accounted for by the choice of ${k}_{trans}$ and ${k}_{met}$. We assume linear kinetics with no dose-dependence, as supported by previous findings (Guo et al. 2021; Frobish et al. 1986).

The excretion into milk is assumed to be continuous, as the cow secretes milk into the udder’s cisterns, which are then periodically emptied during milking. Lastly, it is assumed that the input into the system happens instantaneously; this simplification is supported by the fact that the ${T}_{\mathrm{max}}$ of AFB1 concentration in blood occurred after 35 min, as reported by (Guo et al. 2021). Furthermore, it is assumed that 100% of the AFB1 is absorbed into the system, as it is impossible to distinguish between fraction absorbed and the fraction that is transformed into AFM1 given only blood and milk concentrations; the true fraction unabsorbed is embedded in the ratio of ${k}_{trans}$ and ${k}_{met}$. The model amounts in the animal are described by the following differential equations

1 $$\dot{A}=MA+I$$

with $$A=\left(\begin{array}{c}{A}_{AFB1}\\ {A}_{AFM1}\end{array}\right)$$ containing the amounts of AFB1 and AFM1 in the cow.

2 $$M=\left(\begin{array}{cc}-({k}_{met}+{k}_{trans})& 0\\ {k}_{trans}& -({\mathrm{k}}_{eli}+{k}_{milk}{V}_{milk})\end{array}\right)$$

is the transition matrix and $I$ is the input vector describing the continuous input (only background contamination $I={\left({I}_{bg},0\right)}^{T}$) into the system. The amount of AFM1 in milk at time of milking can be integrated as

3 $${A}_{milk}={\int }_{{t}_{0}}^{{t}_{1}}{k}_{milk}{(t)V}_{milk}(t){A}_{AFM1}(t)dt,$$

where ${t}_{0}$ is the time of the previous milking and ${t}_{1}$ is the time of current milking. Finally, in (Guo et al. 2021) also ct-profiles in blood were available, making it valuable to also predict blood concentrations ${C}_{blood,AFM1}=\frac{{A}_{AFM1}(t)}{V{D}_{AFM1} BW}$ and ${C}_{blood,AFB1}=\frac{{A}_{AFB1}(t)}{V{D}_{AFB1} BW}$, where $V{D}_{AFM1}$ and $V{D}_{AFB1}$ are the volumes of distribution and BW = 500 kg is the body weight, which are also inferred from the data.

### Deriving the model parameters

#### Reparameterization

Four parameters related across multiple studies ${k}_{met}$, ${k}_{trans}$, ${k}_{eli}$ and ${k}_{milk}$ need to be derived. $V{D}_{AFM1}$ and $V{D}_{AFB1}$ are only derived for (Guo et al. 2021). The parameters ${k}_{met}$, ${k}_{trans}$, ${k}_{eli}$ and ${k}_{milk}$ show strong correlations among each other when estimated from concentration data. This correlation is not necessarily a consequence of the biological processes themselves but rather is a consequence of the model structure. Importantly, the correlation is not strictly linear and may not be well described by a multivariate normal distribution. Therefore, these parameters may become numerically unstable and potentially lead to an unrealistically high variance. To address this, the model is reparameterized to derive new parameters that ensure numerical stability in their inference from ct-profiles and reduce sensitivity to small errors in our assumptions. Below, we replace the four original correlated parameters with four new, more independent parameters: (a) steady state transfer rate ($\mathrm{TR})$, (b) mean residence time (MRT), (c) proportion of the MRT spent as parent AFB1 before biotransformation to AFM1, and (d) how much does biotransformation vs. milk excretion contribute to $TR$. Of these parameters, the TR and MRT are directly obtainable from the experimental data without a specific model. The other two parameters are more abstract but indirectly relate the previous parameters to the milk yield. All 4 parameters are dependent on the milk yield and are consequently derived using a reference cow with milk yield ${V}_{milk}$ = 25 L/d, which is roughly in the middle of all studies. Parameters that depend on the reference milk yield have the index 25 to illustrate this. The first new parameter (a) the steady state transfer rate ($T{R}_{25}=TR(25L/d)$), which describes the amount of AFB1 transferred as AFM1 in milk, can be derived as

4 $$T{R}_{25}={F}_{trans}{F}_{excreted,25}$$

with.

5 $${F}_{trans}=\frac{{k}_{trans}}{{k}_{met}+{k}_{trans}},$$

the fraction of eliminated AFB1 which is transformed into AFM1 and

6 $${F}_{excreted,25}={\left.\frac{{k}_{milk}{V}_{milk}}{{k}_{eli}+{k}_{milk}{V}_{milk}}\right|}_{{V}_{milk} = 25\text{ L}/\mathrm{d}},$$

the fraction of eliminated AFM1, which is eliminated via excretion with milk. Furthermore, $T{R}_{25}$ is logit transformed to reflect the fact that $T{R}_{25}\in (\mathrm{0,1})$. The second new parameter is (b) mean residence time ($MR{T}_{25}=MRT(25L/d)$), which describes the mean amount of time a molecule takes from ingestion as AFB1 until excretion via milk as AFM1. This is roughly proportional to the time until steady state and roughly inversely proportional to the average decline rate of AFM1 concentration, which is relatively independent of $T{R}_{25}$ from model structure point of view. The $MR{T}_{25}$ can be calculated as

7 $$MR{T}_{25}={\left.\frac{{k}_{milk}{V}_{milk}{\left({\int }_{0}^{\infty }t{e}^{Mt}{\left(\mathrm{1,0}\right)}^{T}dt\right)}_{AFM1}}{\mathrm{TR}({\mathrm{V}}_{\mathrm{milk}}) }\right|}_{{V}_{milk} = 25\text{ L}/\mathrm{d}}$$

8 $$={\left.\frac{{{k}_{milk}{V}_{milk}\left({M}^{-2}{\left(\mathrm{1,0}\right)}^{T}\right)}_{AFM1}}{\mathrm{TR}({\mathrm{V}}_{\mathrm{milk}}) }\right|}_{{V}_{milk} = 25\text{ L}/\mathrm{d}}$$

9 $$={\left.\frac{1}{{k}_{met}+{k}_{trans}}+\frac{1}{{k}_{eli}+{k}_{milk}{V}_{milk}}\right|}_{{V}_{milk} = 25\text{ L}/\mathrm{d}}$$

10 $$=\frac{1}{{k}_{AFB1}}+\frac{1}{{k}_{AFM\mathrm{1,25}}}$$

with

11 $${k}_{AFB1}={k}_{met}+{k}_{trans}$$

and

12 $${k}_{AFM1,25}={\left.{k}_{eli}+{k}_{milk}{V}_{milk}\right|}_{{V}_{milk} = 25\text{ L}/\mathrm{d}}$$

being the clearance rates of AFB1 and AFM1 respectively. Furthermore, it is assumed that $MR{T}_{25}$ is log normally distributed so that $MR{T}_{25}\in (0,\infty )$. The third (c) and fourth parameters (d) could not be chosen to be insensitive to minor assumption errors. However, they are designed to try to minimize dependence on the first two parameters. The third parameter (c) ${F}_{MR{T}_{25}}$ is the mean fraction of time of the $MR{T}_{25}$ that the aflatoxin scaffold spends as AFB1 in the cow before being transformed into AFM1 and then excreted via milk, i.e.

13 $${F}_{MR{T}_{25}}MR{T}_{25}=\frac{1}{{k}_{AFB1}} .$$

To ensure that ${F}_{MR{T}_{25}}\in (\mathrm{0,1})$ it assumed that it is logit normally distributed. Finally, to ensure the non-negativity of the ${k}_{i}$​, a somewhat more abstract fourth parameter is introduced (d) ${F}_{{\mathrm{TR}}_{25}}$ or the fraction of $log\left({\mathrm{TR}}_{25}\right)$ that is induced due to the fraction of AFB1 transformed into AFM1, i.e.

14 $${F}_{T{R}_{25}}log\left({\mathrm{TR}}_{25}\right)=log\left({F}_{trans}\right)$$

It is assumed that ${F}_{{\mathrm{TR}}_{25}}$ is logit normally distributed, so that ${F}_{{\mathrm{TR}}_{25}}\in (\mathrm{0,1})$. In summary, we assume that $logit\left(T{R}_{25}\right),log\left(MR{T}_{25}\right),logit\left({F}_{MR{T}_{25}}\right)$ and $logit\left({F}_{T{R}_{25}}\right)$ are independently normally distributed. The transformation of these reparametrized forms back to their original form (${k}_{met}$, ${k}_{trans}$, ${k}_{eli}$ and ${k}_{milk}$) is shown in the supplementary section “Deriving the parameters back from the reparameterizations”.

#### Hierarchical Bayesian inference

**Fig. 2:**
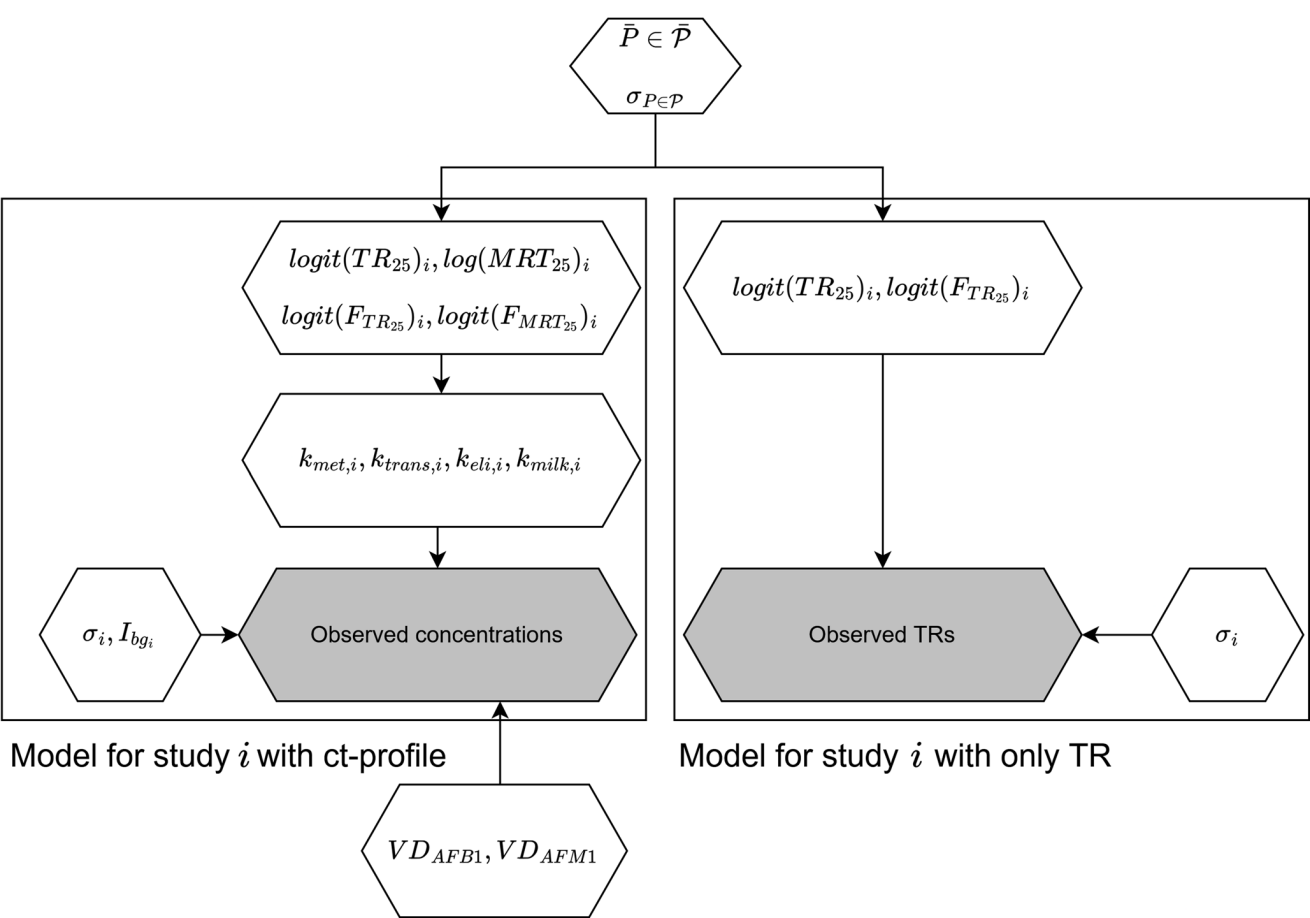
Schematic description of the hierarchical Bayesian inference approach. Here $\mathcal{P}$ represents all reparametrized model parameters $\mathrm{logit}\left({\mathrm{TR}}_{25}\right),\mathrm{log}\left({\mathrm{MRT}}_{25}\right),\mathrm{logit}\left({\mathrm{F}}_{{\mathrm{MRT}}_{25}}\right)$ and $\mathrm{logit}\left({\mathrm{F}}_{{\mathrm{TR}}_{25}}\right)$. All log are natural logarithms

To derive the parameter distributions, a hierarchical Bayesian inference approach is used, as shown schematically in Fig. 2. In this approach, it is assumed that each study has its own parameter distribution, but the meta-parameters of these distributions are themselves drawn from an overarching distribution. The aim is to parameterize this overarching distribution. To do this, each study $i$ with a ct-profile reported is described by an individual set of parameters $logit{\left(T{R}_{25}\right)}_{i},log{\left(MR{T}_{25}\right)}_{i},logit{\left({F}_{MR{T}_{25}}\right)}_{i}$ and $logit{\left({F}_{{\mathrm{TR}}_{25}}\right)}_{i}$. Studies $i$ with only the TR reported are described by only $logit{\left({\mathrm{TR}}_{25}\right)}_{i}$ and $logit{\left({F}_{{\mathrm{TR}}_{25}}\right)}_{i}$ since $log{\left(MR{T}_{25}\right)}_{i}$ and $logit{\left({F}_{MR{T}_{25}}\right)}_{i}$ have no influence on the prediction of the ${\mathrm{TR}}_{25}$ at any milk yield, and are therefore not inferred to avoid nuisance parameters. All parameters $logit{\left(T{R}_{25}\right)}_{i},log{\left(MR{T}_{25}\right)}_{i},logit{\left({F}_{MR{T}_{25}}\right)}_{i}$ and $logit{\left({F}_{T{R}_{25}}\right)}_{i}$ are assumed to be independently normally distributed with mean $\overline{logit\left(T{R}_{25}\right)},\overline{log\left(MR{T}_{25}\right)},\overline{logit\left({F}_{MR{T}_{25}}\right)}$ and $\overline{logit\left({F}_{T{R}_{25}}\right)}$, respectively. Therefore, the log probability for each parameter $P$ is given by

15 $${l}_{mean,P}{\left(\overline{P},{(P}_{i}\right)}_{i\in {\Omega }_{\mathrm{P}}})={\sum }_{{\Omega }_{\mathrm{P}}}-\frac{1}{2}\frac{{{(P}_{i}-\overline{P})}^{2}}{{\sigma }_{P}^{2}}-({|\Omega }_{\mathrm{P}}|+1)\mathrm{log}\left({\upsigma }_{\mathrm{P}}\right),$$

where ${\Omega }_{\mathrm{P}}$ are the studies, where parameter $P$ is inferred and ${\upsigma }_{\mathrm{P}}^{2}$ is the variance of parameter $P$. The last $\mathrm{log}\left({\upsigma }_{\mathrm{P}}\right)$ comes from Jeffreys prior, which we assumed as it represents an uninformative prior. The log probabilities for the study $i$ individual parameters are given by

16 $$\begin{gathered} l_{i} \left( {{\mathcal{P}}_{i} ,I_{{bg,i}} ,VD_{{AFM1}} ,VD_{{AFB1}} } \right) = \sum\limits_{j}^{{n_{i} }} {} \hfill \\ \quad - \frac{{\left( {{\mathrm{log}}\left( {p_{{j,i}} \left( {{\mathcal{P}}_{i} ,I_{{bg,i}} ,VD_{{AFM1}} ,VD_{{AFB1}} } \right)} \right) - log\left( {m_{{j,i}} } \right)} \right)^{2} }}{{2\sigma _{{j,i}}^{2} }} \hfill \\ \quad - \left( {n_{i} + 1} \right)log\left( {\sigma _{i} } \right), \hfill \\ \end{gathered}$$

where ${\mathcal{P}}_{i}$ are the model parameters derived from study $i$. Here, ${p}_{j,i}$ and ${m}_{j,i}$ are the ${n}_{j}$ predicted and measured concentrations at the given time point or the TR. The background contamination is given by ${I}_{bg,i}$ and volumes of distribution $V{D}_{AFM1},V{D}_{AFB1}$ for AFM1 and AFB1. The transfer rate was predicted using the steady state solution of the model given the constant reported milk yield in the study, i.e.,

17 $$\mathrm{TR}({V}_{milk})=k_{milk}V_{milk}{\left(-{M({V}_{milk})}^{-1}{\left(\mathrm{1,0}\right)}^{T}\right)}_{AFM1}$$

18 $$={F}_{trans}\frac{{F}_{excreted,25}}{{F}_{excreted,25}\left(1-\frac{25}{{V}_{milk}}\right)+\frac{25}{{V}_{milk}}}$$

If no background contamination was apparent in the data, i.e., all measurements before dosing are below LOQ, then ${I}_{bg,i}$ was set to 0. Otherwise, if a hint of a background contamination was apparent in the data (Guo et al. 2021) (multiple doses), (Britzi et al. 2013), (Masoero et al. 2007), ${I}_{bg,i}$ was also derived via the inference process, where it is assumed that at the start of the study, the animals are in steady state, i.e., $A\left(0\right)=-{M}^{-1}{\left({I}_{bg,i},0\right)}^{T}$. The parameters $V{D}_{AFM1}$ and $V{D}_{AFB1}$ are only relevant for the study by (Guo et al. 2021), as it is the only study that reported blood concentrations. The ${\sigma }_{j,i}^{2}$ are the respective variances, which in the case that they were reported in the study and more than 8 datapoints were available then ${\sigma }_{i}^{2}={\sigma }_{j,i}^{2}$ was inferred and otherwise ${\sigma }_{j,i}^{2}=0.4$ was assumed, which is a rough conservative estimate of the average study variance. The last $-log({\sigma }_{i})$ comes again from the Jeffreys prior, which we assumed. In summary the log probability to derive the distribution of all parameters is given by

19 $${l}_{total}={\sum }_{P\in \mathcal{P}}{l}_{mean,P}+{\sum }_{i\in\Omega }{l}_{i},$$

where $\mathcal{P}$ is the set of all model parameters and $\Omega$ the set of all studies. Further details can be found in the supplementary section “Data curation”.

#### Implementation of the inference

We performed inference using the PyMC package in Python 3.8.8, employing the NUTS sampler with 40 chains, 1000 tuning steps and 1000 sampling steps (Abril-Pla et al. 2023). The derivative of the log likelihood function was derived using the approx_fprime function of scipy (Virtanen et al. 2020). To reduce autocorrelation, only every 10th sample was retained. To enhance convergence and reduce overflow issues, parameter priors were constrained: $MRT$’s and $\sigma$’s were limited to [0.01, 9.99], while ${F}_{MRT}$’s, ${F}_{TR}$‘s and TR’s were limited to [0.001, 0.999]. More information can be found in the Supplementary section “Implementation of the inference and convergence”.

#### Generating a distribution across studies using Monte Carlo

In order to generate the distribution for simulations including the credible intervals across studies, a Monte Carlo (MC) approach is followed. For each MC sample $j$ and parameter$P\in \mathcal{P}$, a mean ${\overline{P} }_{j}$ from the overarching parameter distribution and a ${\sigma }_{P,j}$ are drawn from the inferred distribution. Definitive samples of this parameter are then generated by randomly drawing from $N(\overline{{P }_{j}} ,{\sigma }_{P,j})$ 10 times. This then represents 10 new, realistic possible sets of parameters derived from potential new studies.

#### Ethical standards

This is a purely in silico study that uses published material. No new animal or human studies were involved (Fig. 2).

### Data availability

All data used in this study is already published and available in each respective publication. The final model code is included as a supplement. The model is also publicly available for interactive calculation in the ConTrans website https://contrans.bfr.bund.de

## Results

**Fig. 3:**
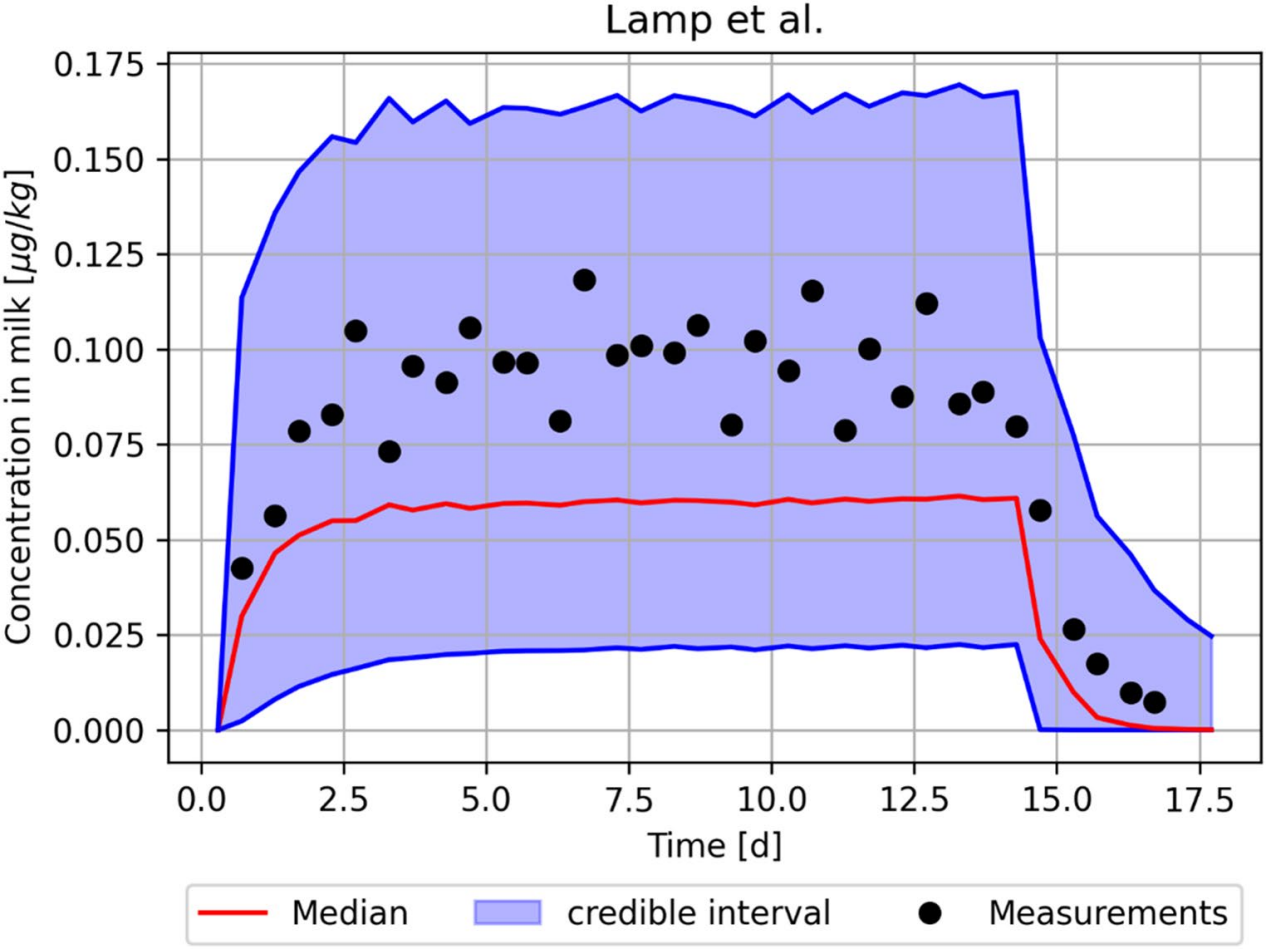
Exemplary plot of the predicted ct-profile for milk compared to measured concentrations in (Lamp et al. 2026) of cow 3549. The median and credible interval are obtained using the Monte Carlo method described

The resulting model and parameters can be found in Supplementary section “Consensus model summary”. To illustrate the predictive model, cow 3549 of (Lamp et al. 2026) is simulated and compared to the experimental data in Fig. 3. As expected, the ct-profiles rise rapidly to a steady state concentration during the assimilation phase and then decline rapidly in the depuration phase. The credible intervals are quite large, ranging at steady state between 0.02 $\mu g/L$ and 0.16 $\mu g/L$, i.e., at steady state the range varies roughly by a factor of 8.

**Fig. 4:**
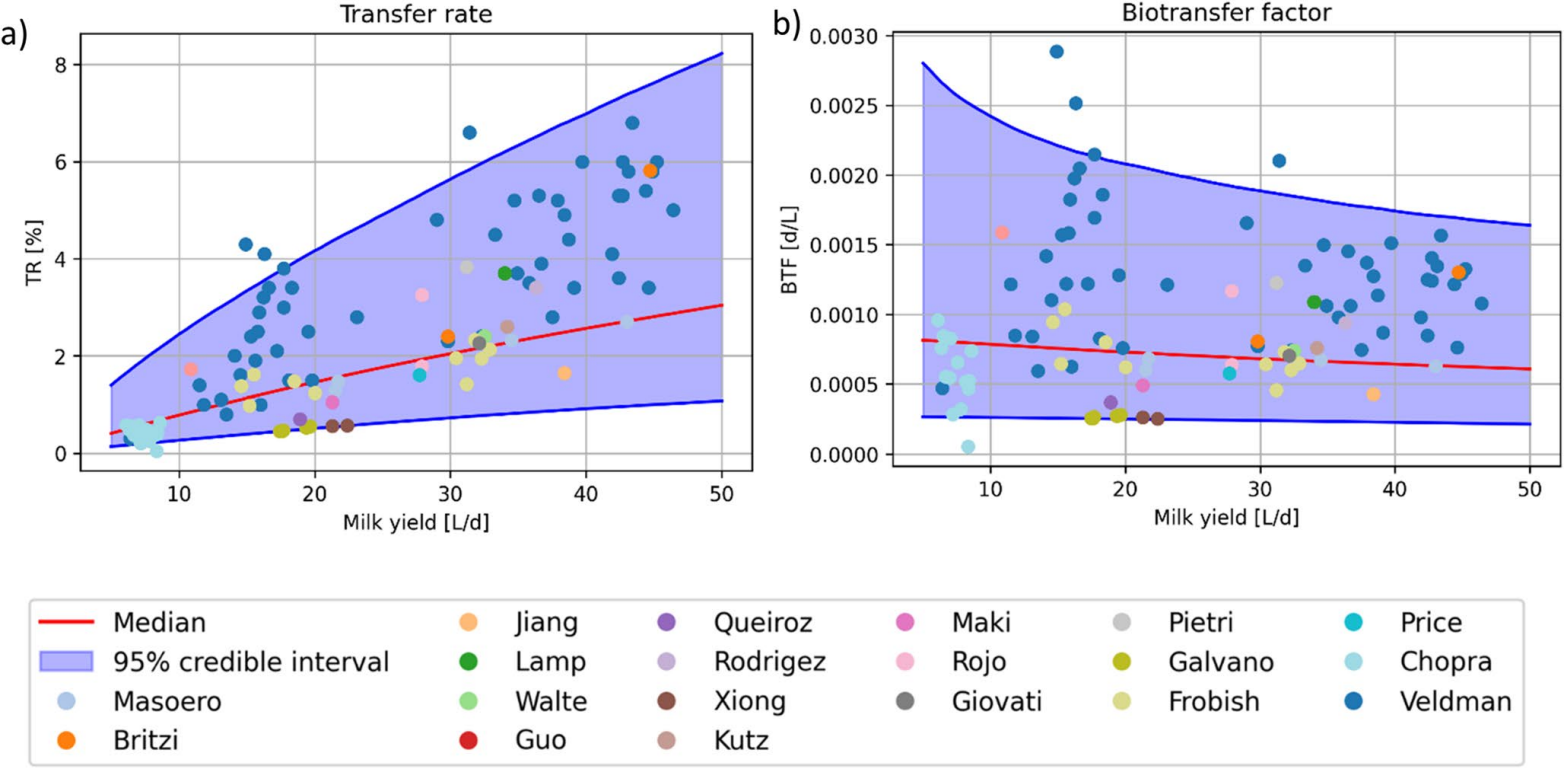
Predicted transfer rate $\mathrm{TR}$ depending on milk yield (lines in a) and BioTransfer Factors $\mathrm{BTF}$ at different milk yields (lines in b) compared to the measurements reported in the respective studies (dots). The median and credible intervals were obtained using the Monte Carlo method described

An estimation of the transfer rates at steady state ($TR({V}_{milk})$) at different milk yields ${V}_{milk}$ can be seen in Fig. 4, where the predictions are compared to the reported TR in the different studies. For all studies most of their $TR({V}_{milk})$ are inside the credible interval. A substantial impact of milk yield on the transfer rates can be observed with a median transfer rate of 0.41% and a 95% credible interval of 0.13–1.40% at 5 L/d, increasing to a median of 3.05% and a 95% credible interval of 1.07–8.19% at 50 L/d. In contrast to TR, the BioTransfer Factor (BTF, Fig. 4) is more stable, starting with a median of 0.0008 d/L and 95% credible interval of 0.0003–0.0028 d/L at 5 L/d, which decreases to a median of 0.0006 d/L and 95% credible interval of 0.0002–0.0016 d/L at 50 L/d.

**Fig. 5:**
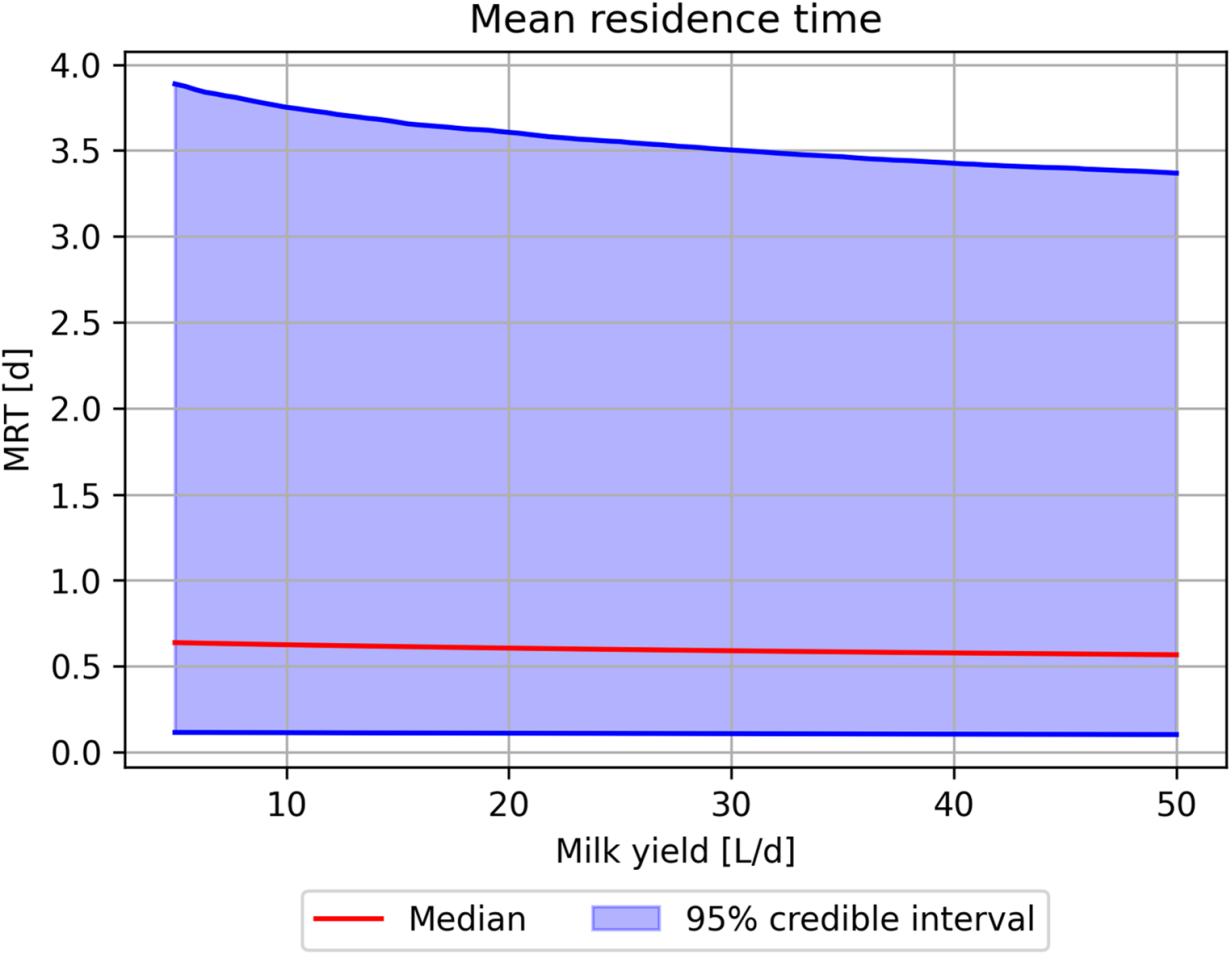
Predicted milk yield-dependent mean residence times ($\mathrm{MRT}({\mathrm{V}}_{\mathrm{milk}})$). The median and credible interval are obtained using the Monte Carlo method described

The mean residence time $MRT({V}_{\mathrm{milk}})$ is not strongly dependent on milk yield ${V}_{milk}$ (Fig. 5). It decreases only slowly with an increased milk yield. It starts with a median 0.64 days (95% Credible Interval CI 0.11–3.95) at 5 L/d, and decreases to a median of 0.57 (95% CI 0.10–3.41) at 50 L/d.

**Fig. 6:**
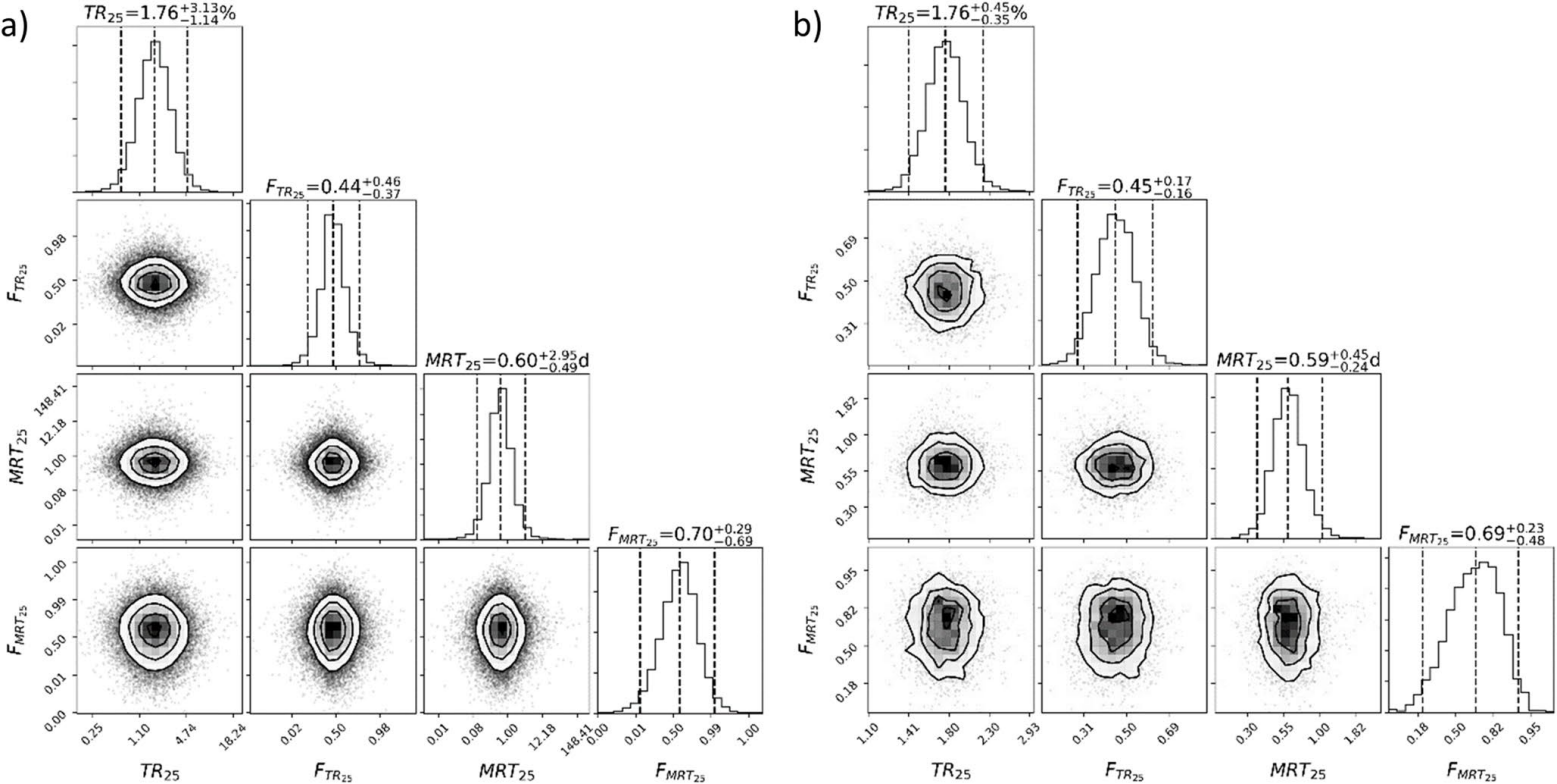
Corner plots of the distribution of the reparametrized model parameters. a) shows the distribution across all studies and b) shows the distribution of the mean. The dashed lines show the [0.025, 0.5, 0.975] quantiles and the title depicts ${\mathrm{X}}_{-\mathrm{Z}}^{+\mathrm{Y}}$, where $\mathrm{X}$ is the median and Y,Z the distance of the median to the upper, lower quantiles respectively. The axis for ${\mathrm{F}}_{{\mathrm{TR}}_{25}}$ and ${\mathrm{F}}_{{\mathrm{MRT}}_{25}}$ are logit transformed; the axes for ${\mathrm{TR}}_{25}$ is logit transformed and multiplied by 100; the axis for ${\mathrm{MRT}}_{25}$ is log transformed. The distribution across studies is derived using the Monte Carlo method described

The resulting reparametrized model parameter distributions are shown in Fig. 6, while the original parameters are shown in Figures S2 and S3. The mean TR and MRT can be derived with a relatively stable value. As expected, the TR and MRT distributions across studies are much wider, but still mostly realistic. However, some rare samples result in unrealistically high values for these parameters, especially the MRT (Zentai et al. 2023). This is due to the smaller number of studies describing the MRT and the heavier-tailed nature of the log-normal distribution. For ${F}_{TR}$, the mean can be derived relatively stably, but the distribution across studies varies almost across the entire spectrum. For ${F}_{MRT}$, the mean varies considerably and the distribution covers the entire possible spectrum with distribution of the variance being mostly limited by the limits set for the sampler (see supplementary section “Implementation of the inference and convergence”), which is probably due to the observed multimodality of this parameter at the edge of its parameter space.

## Discussion

### Mechanistic interpretation

The milk yield-dependent mean residence time MRT (Fig. 5) is relatively flat. If milk excretion were the primary elimination route, then a tenfold increase in milk yield (5–50 L/d) should dramatically decrease MRT (more extraction capacity would mean faster clearance). The flatness of the curve is evidence that milk excretion is not the most important elimination pathway for AFB1 but rather either the direct elimination of AFB1 or the transformation of AFB1 into AFM1 and other metabolites and subsequent elimination via non-milk pathways. This is supported by radiolabeled AFB1 (and metabolites) excretion measurements of only ~ 2% via milk in rats and goats, with most elimination via feces (biliary route) followed by urine (renal route)(Bastaki et al. 2010).

One can observe a sevenfold difference in median TR (0.41%–3.05%) as a consequence of the tenfold difference in milk-yield (5–50 L/d) in Fig. 4. The stronger milk yield-dependence of TR indicates that the kinetics of especially AFM1 inside the animal must be almost unaffected by the milk yield. Consequently, this indicates that the main elimination pathway of especially AFM1 is not the excretion via milk but rather via non-milk pathways. This is because to have an almost linear TR dependence on milk yield, the kinetics of AFM1 must be almost unaffected by milk yield. The fact that there is less increase in median TR (sevenfold) from an increase in milk-yield (tenfold) suggests milk excretion in high-yielding cows gains some importance relative to elimination routes.

### Regulatory implications

The impact of milk yield on BTF (Fig. 4) can be observed, with a median $BTF$ of 0.0008 d/L (95% CI 0.0003–0.0028) at 5 L/d, decreasing to a median of 0.0006 d/L (95% CI 0.0002–0.0016) at 50 L/d. A transfer estimation using the milk yield-dependent BTF and a realistic dry matter intake (DMI, estimated at 23kg 88% DM/d (Spiekers and Schiefler 2021)) reveals that the current EU maximum level for AFB1 in compound feed for dairy cows of 5 µg/kg 88% DM (EU Regulation 574/2011) cannot ensure compliance with the milk AFM1 ML of 0.05 µg/kg across dairy production systems. Model predictions indicate that both low producing and high producing cows would exceed the ML even with compliant feed. This indicates a mismatch in current EU feed and food regulations, which is nevertheless neither unique to Aflatoxin B1/M1 nor surprising. It is not surprising because MLs are typically set using market statistics that are separate for feed and food, with usually no explicit consideration of transfer. As a matter of fact, we estimate that the ML for compound feed for dairy cows should be below 2.65 µg/kg 88% DM in order to be protective for all milk yields in median or below 0.78 µg/kg 88% DM to cover the 95% CI in this model. This is consistent with the recent observation by the German Carry-Over Group that feeding at around half the maximum level (ML) leads to the ML being exceeded in milk in one experiment (BMLEH April 2025).

### Limitations of the model

The observed kinetics and the present model mainly allow us to discriminate the relative importance of milk excretion versus other mechanisms as a group. Despite the inclusion of milk yield as a variable, the range of the 95% CI in all 3 parameters remains rather large (Figs. 4, 5), indicating that other factors not accounted for in this model must have significant impact on transfer. Many influencing factors for this have been proposed, including somatic cell count (Masoero et al. 2007), type of feed (Zentai et al. 2023), breeds (Mins et al. 2021), and lactation stage (Britzi et al. 2013). Physiological changes in high-producing cows, including altered hepatic enzyme expression (Giantin, Rahnasto-Rilla et al. 2019), absorption and rumen microbiota (Zentai, Jozwiak et al. 2023) have been discussed. In this context, a specific hypothesis has been proposed elsewhere that involves diet-induced rumen dysfunction: high-yielding cows receive greater concentrate feed proportions, increasing starch and lowering rumen pH to trigger subacute ruminal acidosis (SARA). SARA impairs the ruminal barrier, potentially increasing AFB1 absorption, while SARA-associated microbiota shifts could alter AFB1 metabolism (BMLEH 2025). Direct measurement of these mechanisms, together with milk yield-dependent feeding studies in dairy cows would be required to test these hypotheses.

The model (Fig. 1) treats AFB1 as if it apparently distributed systemically. In reality, AFB1 is almost entirely hepatically extracted on first pass (Iori et al. 2024). This means that the current model is effectively liver-specific. The apparent “central compartment” is functionally dominated by the liver and portal blood. This issue is more one of model structure interpretation and does not affect the practical model predictions.

Generally, the model assumes that the distribution of animals in the potential use case reflects the distribution of animals in the studies on which this model is based. This assumption is imperfect, however. One potential issue is that some of the studies are quite old, meaning that the genetics of the animals used may no longer be relevant. However, incorporating such a broad range of studies ensures that our predicted credible interval encompasses the most likely scenarios, albeit potentially at the expense of conservatism. The model provides a pragmatic tool for risk analysis in practical farm settings.

## Conclusion

This consensus toxicokinetic model for the transfer of AFB1 as AFM1 in milk provides the first quantitative prediction of milk yield-dependent aflatoxin M1 (AFM1) transfer with explicit uncertainty quantification (credible intervals), directly addressing a critical gap in current regulatory risk analysis. We found a strong milk yield dependence (0.41% median transfer rate in low milk yield vs. 3.05% median in high milk yield). The transfer calculations show that the current EU maximum level (ML) for AFB1 in compound feed for dairy cows is not protective against exceedance of the milk AFM1 ML. The hierarchical Bayesian approach enabled evidence synthesis across 19 feeding studies, overcoming parameter correlations that limited prior deterministic models. The credible intervals quantify the residual prediction uncertainty after accounting for milk yield, including between-study heterogeneity and the effects of unmeasured (and in practical farm settings unmeasurable) biological factors. The substantial residual variability indicates that unmeasured biological factors are important modulators of transfer; direct measurement of these factors in scientific studies coupled with feeding studies would help develop mechanistic understanding. The model nevertheless provides a pragmatic tool for risk assessment and risk management in typical dairy production systems. It is hoped that it can assist regulatory decisions about feed safety, as contamination patterns shift with climate change and feed globalization. The model is made available as computer code in the supplements and on the web as an interactive calculation in the graphical web tool ConTrans.bfr.bund.de.

## Supplementary Information

Below is the link to the electronic supplementary material.

Supplementary Material 1

Supplementary Material 2
