# Supplementary material for: Quantifying milk yield-dependent aflatoxin B1-to-M1 transfer in dairy cows: a Bayesian consensus toxicokinetic model
Source: Arch Toxicol. 2026 Apr 28;100(8):3479–89. doi: 10.1007/s00204-026-04366-3 (PMC13379461; doi:10.1007/s00204-026-04366-3)
Supplement: Supplementary file 1 — Supplementary Material 1 [file 204_2026_4366_MOESM1_ESM.docx]

Supplementary Material

Quantifying Milk Yield-Dependent Aflatoxin B1-to-M1 Transfer in Dairy Cows: A Bayesian Consensus Toxicokinetic Model

*Jan-Louis Moenning*^1^, Jorge Numata^1^*

^1^Department Food and Feed Safety in the Food Chain, German Federal Institute for Risk Assessment (BfR), Max-Dohrn-Strasse 8-10, 10589 Berlin, Germany

* corresponding author: jan-louis.moenning@bfr.bund.de

## Consensus model summary

Table S1: Parameters used in the model. All log are natural logarithms.

| **Variable** | **units** | **Description** | **Equation** |
| --- | --- | --- | --- |
| $V_{milk}$ | L/d | Milk yield |  |
| $A\text{=}\left( \begin{matrix} A_{AFB1} \\ A_{AFM1} \end{matrix} \right)$ | $\mu g$ | Amount of AFB1 and AFM1 in the cow | $A\text{=}MA\text{+}I$ |
| $M$ | 1/d | Transition matrix | $\text{=}\left( \begin{matrix} \text{-}(k_{met}\text{+}k_{trans}) & 0 \\ k_{trans} & \text{-}(k_{eli}\text{+}k_{milk}V_{milk}) \end{matrix} \right)$ |
| $I\text{=}\left( \begin{matrix} I_{bg} \\ 0 \end{matrix} \right)$ | $\mu g/d$ | Continuous input into the cow |  |
| $A_{milk}$ | $\mu g$ | Amount of AFM1 in milk | =$\int_{t_{0}}^{t_{1}} k_{milk}{(t)V}_{milk}(t)A_{AFM1}(t)dt$ |
| $C_{milk}$ | $\mu g/L$ | Concentration of AFM1 in milk | =$\frac{A_{milk}}{V_{milk}}$ |
| $C_{blood,AFB1}$ | $\mu g/kg$ | Concentration of AFB1 in milk | $=\frac{A_{AFB1}}{VD_{AFB1}BW}$ |
| $C_{blood,AFM1}$ | $\mu g/kg$ | Concentration of AFM1 in milk | $=\frac{A_{AFM1}}{VD_{AFM1}BW}$ |
| $BW$ | kg | Body weight of the animal |  |
| $TR_{25}$ | unitless | Transfer rate if milk yield is 25 L/d | $\text{=}F_{trans}F_{excreted,25}$ |
| $F_{TR_{25}}$ | unitless | In a log sense, fraction of the aflatoxins not transferred into milk due to elimination as AFB1 if milk yield is 25 L/d | $=\frac{log\left( F_{trans} \right)}{log\left( TR_{25} \right)}$ |
| $MRT_{25}$ | d | Mean residence time if milk yield is 25 L/d | $\text{=}\frac{1}{k_{AFB1}}\text{+}\frac{1}{k_{AFM1,25}}$ |
| $F_{MRT_{25}}$ | unitless | Fraction of time of the MRT the aflatoxin spends as AFB1 if milk yield is 25 L/d | $\text{=}\frac{1}{k_{AFB1}MRT_{25}}$ |
| $\sigma_{i}^{2}$ | unitless | Variance of measurements of study i (for the log transformed measurements) |  |
| $\sigma_{P}^{2}$ | unitless | Variance of parameter P (for the log or logit transformed parameter) |  |
| ${I_{bg}}_{i}$ | $\mu g/d$ | Background contamination of study i |  |
| $k_{met}$ | 1/d | Elimination rate of AFB1 not transformed into AFM1 | $=k_{AFB1}-k_{trans},$ |
| $k_{trans}$ | 1/d | Biotransformation rate of AFB1 into AFM1 | $=F_{trans}k_{AFB1},$ |
| $k_{eli}$ | 1/d | Elimination rate of AFM1 not excreted via milk | $=k_{AFM1,25}-k_{milk}\cdot25$ |
| $k_{milk}$ | 1/d | Milk proportionality factor for the excretion rate of AFM1 in study i | $=\frac{F_{excreted,25}k_{AFM1,25}}{25}$ |
| $k_{AFB1}$ | 1/d | Elimination rate of AFB1 in study i | ${=k}_{met}\text{+}k_{trans}$ |
| $k_{AFM1,25}$ | 1/d | Elimination rate of AFM1 if milk yield is 25 L/d | ${=k}_{eli}\text{+}k_{milk}\cdot25$ |
| $F_{trans}$ | unitless | Fraction of AFB1 transformed into AFM1 | $=e^{F_{TR_{25}}\log\left( TR_{25} \right)}$ |
| $F_{excreted,25}$ | unitless | Fraction of AFM1 excreted via milk if milk yield is 25 L/d | $=\frac{k_{milk}\cdot25}{k_{eli}+k_{milk}\cdot25}$ |
| $VD_{AFB1}$ | unitless | Volume of distribution of AFB1 |  |
| $VD_{AFM1}$ | unitless | Volume of distribution of AFM1 |  |
| $TR(V_{milk})$ | unitless | Milk yield dependent transfer rate | $\text{=}F_{trans}F_{excreted,V_{milk}}$ |
| $BTF(V_{milk})$ | d/L | Milk yield-dependent bio transfer factor | $=\frac{TR\left( V_{milk} \right)}{V_{milk}}$ |
| $MRT(V_{milk})$ | d | Milk yield-dependent mean residence time | $=\frac{1}{k_{AFB1}}\text{+}\frac{1}{k_{AFM1,V_{milk}}}$ |

## The credible intervals reflect the experimental data distribution

We used the model to predict the milk concentration-time (ct-profile) for each study and compared the prediction to the experimentally obtained milk concentrations. Table S2 was generated as a sanity check of the resulting credible intervals. Table S2 reflects the fraction of datapoints inside the 95% CI and 66% CI, respectively. No study had a majority (>50%) of its data points outside the 95% CI. This is not surprising, as the credible interval describes the distribution across studies and therefore only roughly 1/20 studies should have a large proportion of datapoints outside the credible interval. As an additional sanity check, we also consider the 66% credible interval; 12 studies have a majority (>50%) of data points inside the 66% credible interval and 8 studies have a minority (≤50%) of datapoints outside of the 66% credible interval. This is in line with the fact that we would expect 13.33/20 to be inside the credible interval, again a sign that we derived a realistic distribution.

Table S2: Performance of the overarching distribution of model for predicting the milk concentrations in the individual studies by showing the fraction of data points inside the credible interval (CI). If the prediction was at 0 as no contamination was yet assumed, the data point is neglected.

| **Reference** | **#Datapoints in 95% CI / #Datapoints** | **#Datapoints in 66% CI / #Datapoints** |
| --- | --- | --- |
| (Walte et al. 2022) | 129/130 | 116/130 |
| (Lamp et al. 2026) | 131/131 | 68/131 |
| (Guo et al. 2021) (multi) | 60/63 | 40/63 |
| (Guo et al. 2021) (single) | 9/11 | 2/11 |
| (Britzi et al. 2013) | 18/18 | 12/18 |
| (Jiang et al. 2018) | 8/9 | 8/9 |
| (Masoero et al. 2007) | 66/66 | 63/66 |
| (Queiroz et al. 2012) | 3/3 | 0/3 |
| (Rodrigues et al. 2019) | 5/5 | 5/5 |
| (Xiong et al. 2015) | 6/6 | 2/6 |
| (Kutz et al. 2009) | 1/1 | 1/1 |
| (Pietri et al. 2009) | 1/1 | 0/1 |
| (Giovati et al. 2014) | 1/1 | 1/1 |
| (Rojo Martinez et al. 2014) | 2/2 | 1/2 |
| (Maki et al. 2016) | 1/1 | 1/1 |
| (Galvano et al. 1996) | 5/6 | 0/6 |
| (Frobish et al. 1986) | 10/10 | 10/10 |
| (Price et al. 1985) | 1/1 | 1/1 |
| (Chopra et al. 1999) | 13/14 | 11/14 |
| (Veldman et al. 1992) | 48/52 | 20/52 |

## Deriving the parameters back from the reparameterizations

The aim of this section is to derive the original model parameters $k_{trans}, k_{met}, k_{eli},$ $k_{milk}\in\left( 0,\infty\right)$ from the reparametrized $TR_{25},F_{TR_{25}},F_{MRT_{25}}\in(0,1)$ and $MRT_{25}\in(0,\infty)$, where

| $TR_{25}=F_{trans}F_{excreted,25},$ | (S1) |
| --- | --- |
| $F_{TR_{25}}=\frac{\log\left( F_{trans} \right)}{log(TR_{25})}$ | (S2) |
| $MRT_{25}=\frac{1}{k_{AFB1}}+\frac{1}{k_{AFM1,25}},$ | (S3) |
| $F_{MRT_{25}}=\frac{1}{k_{AFB1}MRT_{25}}$ | (S4) |

with

| $F_{trans}=\frac{k_{trans}}{k_{AFB1}},$ | (S5) |
| --- | --- |
| $F_{excreted,25}=\frac{k_{milk}V_{milk}}{k_{AFM1,25}}$ | (S6) |

and

| $k_{AFB1}=k_{met}+k_{trans},$ | (S7) |
| --- | --- |
| $k_{AFM1}=\left. k_{eli}+k_{milk}V_{milk} \right\vert_{V_{milk}=25 L/d}.$ | (S8) |

First using equation (S1) and (S2) we obtain

| $F_{trans}=e^{F_{TR_{25}}\log\left( TR_{25} \right)}$ | (S9) |
| --- | --- |
| $F_{excreted,25}=\frac{TR_{25}}{F_{trans}}.$ | (S10) |

Similarly using equations (S3) and (S4) one gets

| $k_{AFB1}=\frac{1}{F_{MRT_{25}}MRT_{25}},$ | (S11) |
| --- | --- |
| $k_{AFM1,25}=\frac{1}{MRT_{25}-\frac{1}{k_{AFB1}}}.$ | (S12) |

Then using equation (S5) and (S6) results in

| $k_{trans}=F_{trans}k_{AFB1},$ | (S13) |
| --- | --- |
| $k_{milk}=\left. \frac{F_{excreted,25}k_{AFM1,25}}{V_{milk}} \right\vert_{V_{milk}=25 L/d}.$ | (S14) |

Finally, from equations (S7) and (S8) follows

| $k_{met}=k_{AFB1}-k_{trans},$ | (S15) |
| --- | --- |
| $k_{eli}=\left. k_{AFM1,25}-k_{milk}V_{milk} \right\vert_{V_{milk}=25 L/d}.$ | (S16) |

## Data curation

### Walte study

For this study (Walte et al. 2022) the raw data containing the concentration of aflatoxin M1 at each milking time (morning and evening separately) was directly available. This also includes the amount of milk excreted each milking time. As this study analysed the effectiveness of adding binder to the feed to potential decrease the amount of AFB1 absorbed only the measurements before the binder was added were used for model calibration, i.e. until day 8.

### Lamp study

For this study (Lamp et al. 2026) the raw data containing the concentration of aflatoxin M1 at each milking time (morning and evening separately) was directly available and fully utilised. This also includes the amount of milk excreted each milking time.

### Guo-multi study

For this study (Guo et al. 2021) the raw data containing the concentration of aflatoxin M1 at each day (morning and evening milk were pooled) was directly available and fully utilised. This also includes the amount of milk excreted each milking time.

### Guo-single study

For this study (Guo et al. 2021) raw data describing the average concentration in milk of AFM1 and concentrations in blood of AFM1 and AFB1 were available together with their standard deviation at each measurement point. However, the animal specific measurements were not available but only averages across animals and their standard deviation. As concentration averages and standard deviation were not derived under log transformation, they were adjusted using the formulas described in section “Converting mean and variance under log transformation” to be the mean and standard deviation under log transformation. Furthermore, no milk yield specific to the single administration study was reported, instead we assumed it to be the same as the average rate of the multi-dosage study of 10.9 L/d. Lastly as the model used here assumes instantaneous absorption but the blood data clearly shows most the absorption happens during the first hour all data points less than one hour after administration were ignored to not further complicate the model.

### Britzi study

For this study (Britzi et al. 2013) no raw data was available, therefore concentration in daily milk at the different time points and their respective standard deviation were extracted from Figure 2 of the main text. The mean and standard deviation were then used to derive the mean and variance under log transformation using the algorithm described in section “Converting mean and variance under log transformation”. $\sigma_{i}$ was then divided by $\surd6$ to account for the fact that 6 animals were used to derive the mean. Milk yield per measurement time point was not available but the average milk yield in mid and late lactation was reported and used instead as constant during each supplementation phase respectively.

### Jiang study

For this study (Jiang et al. 2018) no raw data was available, therefore concentration in milk at the different time points and their respective standard deviation (converted from their confidence intervals) were extracted from Figure 1 of the main text. Only the data where only AFB1 was administered (labeled with “T”) was used for our model parametrization. The mean and standard deviation were then used to derive the mean and variance under log transformation using the algorithm described in section “Converting mean and variance under log transformation”. $\sigma_{i}$ was then divided by $\surd6$ to account for the fact that 6 animals were used to derive the mean. Milk yield per measurement time point was not available but the average milk yield in mid and late lactation was reported and used instead as constant.

### Masoero study

For this study (Masoero et al. 2007) no raw data was available, therefore concentration in milk were extracted from Figure 1 of the main text. However, no standard deviation or something similar were available so each ct-profile was treated as a separate single animal. As more than 8 samples were available $\sigma_{i}$ could be inferred. Milk yield at each time was graphically reported in the study but for simplicity it was assumed constant for each group.

### Queiroz study

For this study (Queiroz et al. 2012) no raw data was available, therefore concentration in milk were extracted from Figure 1 of the main text, which only depicted the depuration phase. Only the data where only AFB1 was administered (labeled with “T”) was used for our model parametrization. Day 12 of measurements was ignored as it was not possible to scrape this data reliably in log sense due to its proximity to 0. The mean and standard deviation were then used to derive the mean and variance under log transformation using the algorithm described in section “Converting mean and variance under log transformation”. $\sigma_{i}$ was then divided by $\surd2$ to account for the fact that 2 animals were used to derive the mean. Milk yield per measurement time point was not available but the average milk yield in mid and late lactation was reported and used instead as constant.

### Rodrigues study

For this study (Rodrigues et al. 2019) no raw data was available, therefore concentration in milk were extracted from Figure 1 of the main text, which only depicted the depuration phase. Only the data where only AFB1 was administered (labeled with “PC”) was used for our model parametrization. Measurements for day 10 were ignored as it was not possible to scrape this data reliably in log sense due to their proximity to 0. First the same value was converted into the standard deviation by multiplying by $\surd8$ for the 8 animals used. The mean and standard deviation were then used to derive the mean and variance under log transformation using the algorithm described in section “Converting mean and variance under log transformation”. $\sigma_{i}$ was then divided by $\surd8$ to account for the fact that 8 animals were used to derive the mean. Milk yield per measurement time point was not available but the average milk yield in mid and late lactation was reported and used instead as constant.

### Xiong study

For this study (Xiong et al. 2015) no raw data was available, therefore concentration in milk were extracted from Figures of the main text, which only depicted the depuration phase. Only the data where only AFB1 was administered (labeled with “CON”) was used for our model parametrization. Day 14 of measurements was ignored as it was not possible to scrape this data reliably in log sense due to its proximity to 0. Milk yield per measurement time point was not available but the average milk yield in mid and late lactation was reported and used instead as constant. No SD or similar was reported therefore a SD of 0.4 was assumed with only 1 measurement per value therefore $\sigma_{i}=0.4$.

### Kutz study

For this study (Kutz et al. 2009) only TR was reported and only the data where AFB1 alone administration was used. The pooled SEM value was taken to derive the standard deviation by multiplying it by $\surd12$ to account for the 2 measurements for each of 6 animals. The mean and standard deviation was then used to derive the mean and variance under log transformation using the algorithm described in section “Converting mean and variance under log transformation”. $\sigma_{i}$ was then again divided by $\surd12$ to account for the number of measurement points.

### Pietri study

For this study (Pietri et al. 2009) only TR was reported and only the data where AFB1 alone administration was used (‘CTR’). The pooled SEM value was taken to derive the standard deviation by multiplying it by $\surd3$ to account for the averaging TR of 3 distinct weeks. The mean and standard deviation was then used to derive the mean and variance under log transformation using the algorithm described in section “Converting mean and variance under log transformation”. $\sigma_{i}$ was then again divided by $\surd3$ to account for the number of measurement points. Here it should be noted that 3 presents more of a lower estimate for the amount data points used to derive the average as no better estimate was available.

### Giovati study

For this study (Giovati et al. 2014) a ct-profile was reported but the milking and feeding times were not reported. Therefore, only TR of the unvaccinated animals were used. The mean and standard deviation was then used to derive the mean and variance under log transformation using the algorithm described in section “Converting mean and variance under log transformation”. $\sigma_{i}$ was then divided by $\surd24$ to account for the 4 measurements for each of 6 animals.

### Rojo study

For this study (Rojo Martinez et al. 2014) only TR was reported for 2 different study setups of which only the data considering only AFB1 administration were used. The standard deviation was extracted from the figures. The mean and standard deviation was then used to derive the mean and variance under log transformation using the algorithm described in section “Converting mean and variance under log transformation”. $\sigma_{i}$ was then again divided by $\surd8$ for the first study and by $\surd2$ to account for the number of measurement points with 2 measurements per animal (4 and 1).

### Maki study

For this study (Maki et al. 2016) only TR was reported and only the data where AFB1 alone administration was used (‘AFD’). The pooled SEM value was taken to derive the standard deviation by multiplying it by $\surd15$ to account for the 15 animals used. The mean and standard deviation was then used to derive the mean and variance under log transformation using the algorithm described in section “Converting mean and variance under log transformation”. The result was then again divided by $\surd15$ to account for the number of measurement points.

### Galvano study

For this study (Galvano et al. 1996) only TR was reported and only the data without sorbent were used. Milk yields and TRs (=Carry over) were taken from their Table 1. No SD or similar was reported therefore a SD of 0.4 was assumed but a total of 4 measurements per reported data point were reported resulting in $\sigma_{i}=0.4/\surd4$.

### Frobish study

For this study (Frobish et al. 1986) average ct-profiles per dosage group were shown but the milk yield in each dosage group varied drastically. Therefore, only the TR together with their respective milk yields reported in their Table 3 and Table 4 were used. No SD or similar was reported but more than 8 data points were available therefore $\sigma_{i}$ was inferred.

### Price study

For this study (Price et al. 1985) ct-profiles are available but the precise time of feeding and milking were not clear. Therefore, only the average TR reported in the text is used. However, for this no variance was reported therefore a SD of 0.4 was assumed but a total of 49 samples for 90 animals were reported resulting in a variance of $\sigma_{i}=0.4/\surd4410$.

### Chopra study

For this study (Chopra et al. 1999) only TR was reported and only the data without sorbent were used. Milk yields and TRs were taken from their Table 2 using all 5 trials. No SD or similar was reported but more than 8 data points were available therefore $\sigma_{i}$ was inferred.

### Veldmann study

For this study (Veldman et al. 1992) only TR was reported and only the data without sorbent were used. Milk yields and TRs were taken from their Table 2 and 4. No SD or similar was reported but more than 8 data points were available therefore $\sigma_{i}$ was inferred.

### Converting mean and variance under log transformation

Assume a random variable $X$ with $\log\left( X \right)\sim N(\log\left( \mu_{log} \right), \sigma_{log}^{2})$ but only the mean $\mu_{X}=E[X]$ and its variance $\sigma_{X}^{2}=E[\left( X-\mu_{X} \right)^{2}]$ are given, then it shown in (Mood 1950) in chapter 3.5 that

| $\mu_{\log}=\frac{\mu_{X}}{\sqrt{1+\left( \frac{\sigma_{X}}{\mu_{X}} \right)^{2}}}$ | (S17) |
| --- | --- |
| $\sigma_{log}=\sqrt{log\left( 1+\left( \frac{\sigma_{X}}{\mu_{X}} \right)^{2} \right)}.$ | (S18) |

## Implementation of the inference and convergence

During the sampling process, the sampler encountered 129 divergences (<0.33% of all samples), the cause of the divergence is most likely due overflow error in the computation in which case the sampler assumes a loglikelihood of -100000 and can get stuck there. This was also the motivation behind introducing the limits describes above as it decreased the number of divergences drastically.

The Gelman–Rubin statistic $(\hat{r}$), Bulk and tail Effective Sample Size (${ESS}_{bulk}, {ESS}_{tail}$) of the samples as well as their quantiles are shown in Table S3. $\hat{r}$ is a way to **diagnose** convergence of Markov chain Monte Carlo (MCMC) simulations by comparing multiple chains run in parallel. Ideally, $\hat{r}\approx1$ means that the chains are mixing similarly and likely have converged; values below about 1.05 are often used as a practical threshold; higher values mean there is still more variation between chains than within, suggesting lack of convergence or insufficient run length. The ESS tells us how many independent samples our correlated Monte Carlo draws are “really worth” in terms of information content. ${ESS}_{bulk}$focuses on the center of the distribution and ${ESS}_{tail}$ on the edges of the distribution. Larger ESS means more precise estimates and better mixing. This is important because in MCMC, successive draws are correlated (autocorrelation). Values above 400 are often desirable. If ESS is much smaller than 400, this might indicate that the distribution is not well represented by our samples. However, $\hat{r}$ and ESS are both not conclusive diagnostics and performance should be interpreted alongside trace plots. In our case most parameters have $\hat{r}$ ≲ 1.05 and ${ESS}_{bulk}, {ESS}_{tail}>400$ but there are some exceptions. Especially the parameters associated to $F_{MRT}$ often have significantly problematic values. This is due to the fact that the distribution of $F_{MRT}$ for some studies is multimodal, which often prevents proper mixing of the chains (Figure S1). The rest of the parameters with problematic values seem also to be associated to the multimodality of $F_{MRT_{25}}$, as it causes (especially MRT) to have a different distribution in the chains depending on which modality the $F_{MRT}$ is in. However, inside each modality, the chains seem to be often well mixed.
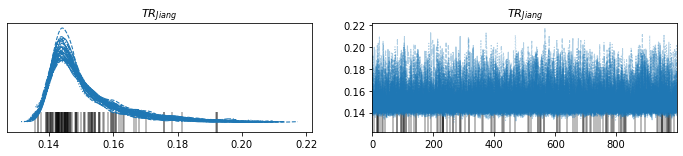

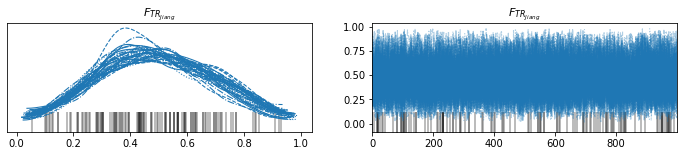

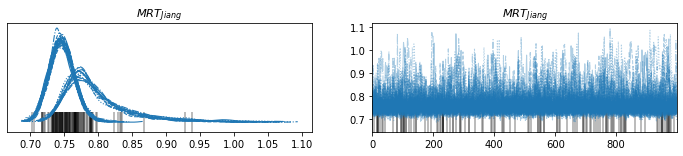

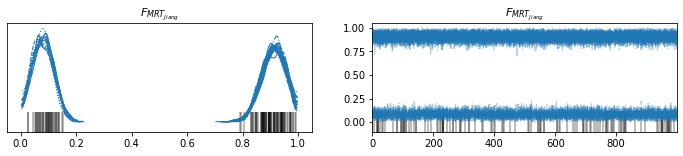


Figure S1: Exemplary plot of the traces (n=40) and their distribution for the parameters derived from (Jiang, Ogunade et al. 2018) using arviz package in python. The black lines at the bottom indicate divergences. It was chosen due to its pronounced multimodality of the $F_{MRT_{25}}$ parameter.

Table S3: Gelman–Rubin statistic ($\hat{r})$, Bulk and tail Effective Sample Size (${ESS}_{bulk}, {ESS}_{tail}$) as well as quantile values of each parameter.

| Parameter | $q_{0.025}$ | $q_{0.5}$ | $q_{0.975}$ | $\hat{r}$ | ${ESS}_{bulk}$ | ${ESS}_{tail}$ |
| --- | --- | --- | --- | --- | --- | --- |
| $TR_{Masoero}$ | 0.012 | 0.013 | 0.015 | 1 | 28810 | 26319 |
| ${F_{TR}}_{Masoero}$ | 0.128 | 0.422 | 0.678 | 1 | 34274 | 21180 |
| $MRT_{Masoero}$ | 0.386 | 0.586 | 0.788 | 1 | 33842 | 18401 |
| $F_{MRT_{Masoero}}$ | 0.011 | 0.938 | 0.993 | 1.16 | 190 | 882 |
| $TR_{Britzi}$ | 0.020 | 0.022 | 0.024 | 1 | 29104 | 28395 |
| $F_{TR_{Britzi}}$ | 0.061 | 0.252 | 0.472 | 1 | 16725 | 22331 |
| $MRT_{Britzi}$ | 0.554 | 0.639 | 0.734 | 1 | 48433 | 31050 |
| $F_{MRT_{Britzi}}$ | 0.312 | 0.508 | 0.718 | 1.01 | 6168 | 27461 |
| $TR_{Jiang}$ | 0.014 | 0.015 | 0.018 | 1 | 9279 | 10787 |
| $F_{TR_{Jiang}}$ | 0.135 | 0.484 | 0.859 | 1 | 9378 | 10774 |
| $MRT_{Jiang}$ | 0.715 | 0.756 | 0.882 | 1.26 | 111 | 484 |
| $F_{MRT_{Jiang}}$ | 0.028 | 0.880 | 0.980 | 1.6 | 67 | 474 |
| $TR_{Lamp}$ | 0.027 | 0.029 | 0.030 | 1.01 | 2252 | 25830 |
| $F_{TR_{Lamp}}$ | 0.059 | 0.245 | 0.463 | 1.05 | 475 | 3106 |
| $MRT_{Lamp}$ | 0.744 | 0.780 | 0.819 | 1.09 | 277 | 1179 |
| $F_{MRT_{Lamp}}$ | 0.004 | 0.974 | 0.997 | 1.6 | 67 | 454 |
| $TR_{Walte}$ | 0.018 | 0.020 | 0.022 | 1.01 | 3719 | 4581 |
| $F_{TR_{Walte}}$ | 0.358 | 0.864 | 0.969 | 1.01 | 2662 | 2219 |
| $MRT_{Walte}$ | 0.447 | 0.552 | 0.668 | 1.01 | 6411 | 7227 |
| $F_{MRT_{Walte}}$ | 0.189 | 0.949 | 0.993 | 1.01 | 3254 | 980 |
| $TR_{Guo}$ | 0.020 | 0.026 | 0.031 | 1 | 20701 | 20658 |
| $F_{TR_{Guo}}$ | 0.136 | 0.488 | 0.839 | 1 | 18836 | 21717 |
| $MRT_{Guo}$ | 0.056 | 0.145 | 0.260 | 1 | 25801 | 16182 |
| $F_{MRT_{Guo}}$ | 0.113 | 0.574 | 0.949 | 1.02 | 1289 | 22071 |
| $TR_{GuoSingle}$ | 0.026 | 0.031 | 0.034 | 1 | 10473 | 7421 |
| $F_{TR_{GuoSingle}}$ | 0.082 | 0.353 | 0.717 | 1 | 8749 | 7308 |
| $MRT_{GuoSingle}$ | 1.173 | 1.251 | 1.312 | 1 | 13583 | 9932 |
| $F_{MRT_{GuoSingle}}$ | 0.615 | 0.655 | 0.707 | 1 | 15710 | 11589 |
| $TR_{Queiroz}$ | 0.007 | 0.008 | 0.009 | 1 | 32337 | 22840 |
| $F_{TR_{Queiroz}}$ | 0.117 | 0.434 | 0.795 | 1 | 26506 | 21523 |
| $MRT_{Queiroz}$ | 0.180 | 0.246 | 0.350 | 1 | 42635 | 29866 |
| $F_{MRT_{Queiroz}}$ | 0.045 | 0.739 | 0.976 | 1.04 | 695 | 5221 |
| $TR_{Rodrigues}$ | 0.019 | 0.020 | 0.024 | 1 | 18003 | 15514 |
| $F_{TR_{Rodrigues}}$ | 0.144 | 0.472 | 0.853 | 1 | 16090 | 15929 |
| $MRT_{Rodrigues}$ | 0.587 | 0.690 | 0.817 | 1 | 13511 | 22404 |
| $F_{MRT_{Rodrigues}}$ | 0.060 | 0.745 | 0.964 | 1.04 | 721 | 6403 |
| $TR_{Xiong}$ | 0.006 | 0.008 | 0.012 | 1 | 33350 | 29524 |
| $F_{TR_{Xiong}}$ | 0.128 | 0.453 | 0.833 | 1 | 32699 | 24350 |
| $MRT_{Xiong}$ | 0.615 | 0.876 | 1.514 | 1 | 27354 | 19004 |
| $F_{MRT_{Xiong}}$ | 0.062 | 0.594 | 0.962 | 1.03 | 754 | 6060 |
| $\sigma_{Masoero}$ | 0.263 | 0.313 | 0.379 | 1 | 42863 | 29883 |
| $\sigma_{Lamp}$ | 0.178 | 0.200 | 0.228 | 1 | 46199 | 29069 |
| $\sigma_{Walte}$ | 0.321 | 0.364 | 0.422 | 1 | 12128 | 15482 |
| $\sigma_{Guo}$ | 0.305 | 0.363 | 0.444 | 1 | 44219 | 29480 |
| $\sigma_{Frobish}$ | 0.137 | 0.213 | 0.381 | 1 | 31606 | 24327 |
| $\sigma_{Chopra}$ | 0.529 | 0.744 | 1.178 | 1 | 43846 | 25929 |
| $\sigma_{Veldman}$ | 0.315 | 0.380 | 0.465 | 1 | 48993 | 27611 |
| $TR_{Kutz}$ | 0.018 | 0.020 | 0.024 | 1 | 28320 | 22026 |
| $F_{TR_{Kutz}}$ | 0.130 | 0.457 | 0.843 | 1 | 22433 | 22516 |
| $TR_{Maki}$ | 0.011 | 0.012 | 0.014 | 1 | 43365 | 30060 |
| $F_{TR_{Maki}}$ | 0.126 | 0.452 | 0.835 | 1 | 32287 | 24812 |
| $TR_{Rojo}$ | 0.021 | 0.024 | 0.027 | 1 | 46905 | 30807 |
| $F_{TR_{Rojo}}$ | 0.131 | 0.463 | 0.844 | 1 | 30479 | 24839 |
| $TR_{Giovati}$ | 0.013 | 0.019 | 0.027 | 1 | 48007 | 29490 |
| $F_{TR_{Giovati}}$ | 0.134 | 0.466 | 0.847 | 1 | 29613 | 26241 |
| $TR_{Pietri}$ | 0.029 | 0.032 | 0.036 | 1 | 30693 | 23721 |
| $F_{TR_{Pietri}}$ | 0.128 | 0.456 | 0.831 | 1 | 22993 | 23042 |
| $TR_{Galvano}$ | 0.006 | 0.007 | 0.008 | 1 | 46083 | 29976 |
| $F_{TR_{Galvano}}$ | 0.122 | 0.431 | 0.799 | 1 | 32616 | 25977 |
| $TR_{Frobish}$ | 0.015 | 0.017 | 0.020 | 1 | 36539 | 23315 |
| $F_{TR_{Frobish}}$ | 0.187 | 0.603 | 0.860 | 1 | 13521 | 25380 |
| $TR_{Price}$ | 0.014 | 0.015 | 0.015 | 1 | 16475 | 12830 |
| $F_{TR_{Price}}$ | 0.130 | 0.459 | 0.839 | 1 | 15928 | 13360 |
| $TR_{Chopra}$ | 0.008 | 0.012 | 0.018 | 1 | 37150 | 27802 |
| $F_{TR_{Chopra}}$ | 0.118 | 0.422 | 0.778 | 1 | 32396 | 25314 |
| $TR_{Veldman}$ | 0.028 | 0.031 | 0.034 | 1 | 46789 | 29886 |
| $F_{TR_{Veldman}}$ | 0.098 | 0.348 | 0.590 | 1 | 32522 | 20729 |
| ${I_{bg}}_{Britzi}$ | 12.896 | 15.800 | 19.664 | 1 | 29199 | 28928 |
| ${I_{bg}}_{Guo}$ | 234.148 | 292.515 | 369.323 | 1 | 40621 | 26557 |
| ${I_{bg}}_{Masoero}$ | 16.063 | 20.015 | 25.526 | 1 | 27604 | 27498 |
| $VD_{AFB1}$ | 16.741 | 18.234 | 19.867 | 1 | 40710 | 31901 |
| $VD_{AFM1}$ | 2.073 | 8.530 | 22.046 | 1 | 8739 | 7360 |
| $\bar{TR}$ | 0.014 | 0.018 | 0.022 | 1 | 39120 | 26169 |
| $\bar{F_{TR}}$ | 0.285 | 0.449 | 0.621 | 1.01 | 8238 | 20118 |
| $\bar{MRT}$ | 0.355 | 0.593 | 1.048 | 1 | 28914 | 19899 |
| $\bar{F_{MRT}}$ | 0.212 | 0.692 | 0.925 | 1.3 | 100 | 321 |
| $\sigma_{TR}$ | 0.132 | 0.251 | 0.544 | 1 | 38258 | 27523 |
| $\sigma_{F_{TR}}$ | 0.086 | 1.044 | 3.377 | 1.02 | 1823 | 1386 |
| $\sigma_{MRT}$ | 0.182 | 0.528 | 2.041 | 1 | 23431 | 18314 |
| $\sigma_{F_{MRT}}$ | 1.281 | 4.901 | 9.541 | 1.11 | 215 | 615 |

### Distribution of parameters


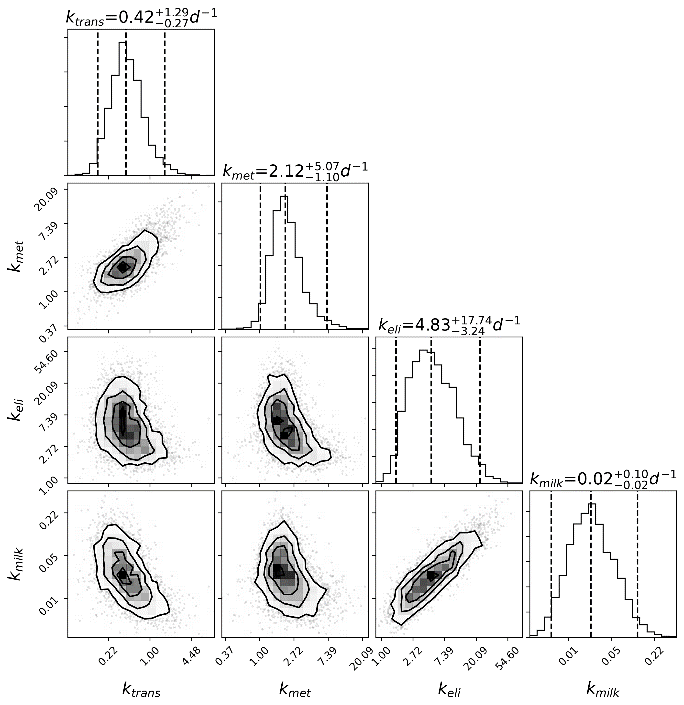

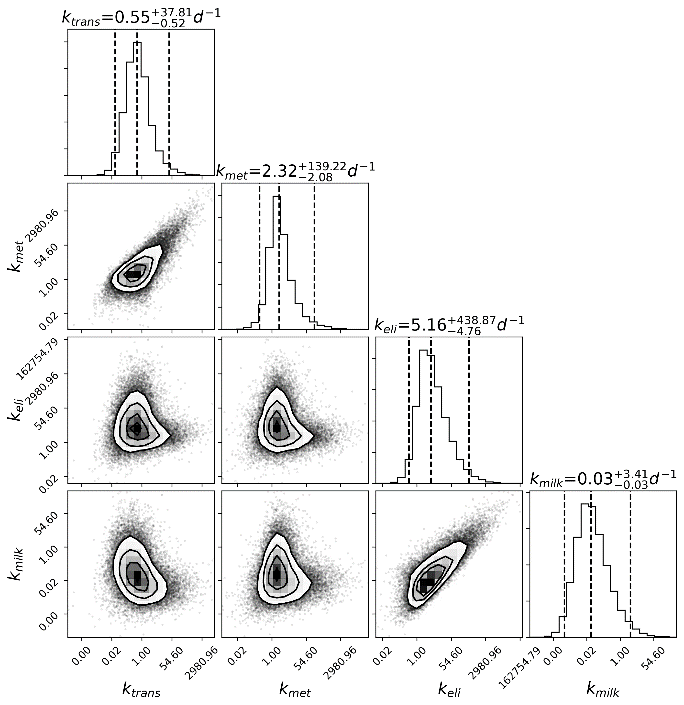


b)

a)

Figure S2: Corner plots of the distribution of the original parameters. a) shows the distribution across all studies and b) shows the distribution of the mean. The dashed lines show the [0.025, 0.5, 0.975] quantiles and the title depicts $X_{-Z}^{+Y}$, where $X$ is the median and Y,Z the distance of the median to the upper, lower quantiles respectively. All axis are log transformed.

In Figure S2 one can observe that already the mean values of the original parameter vary across several orders of magnitude for all parameters, which is in contrast to reparametrized parameters where at least some of them could be stably derived. For the distribution across all studies all parameters, all 4 parameters vary many orders of magnitude.


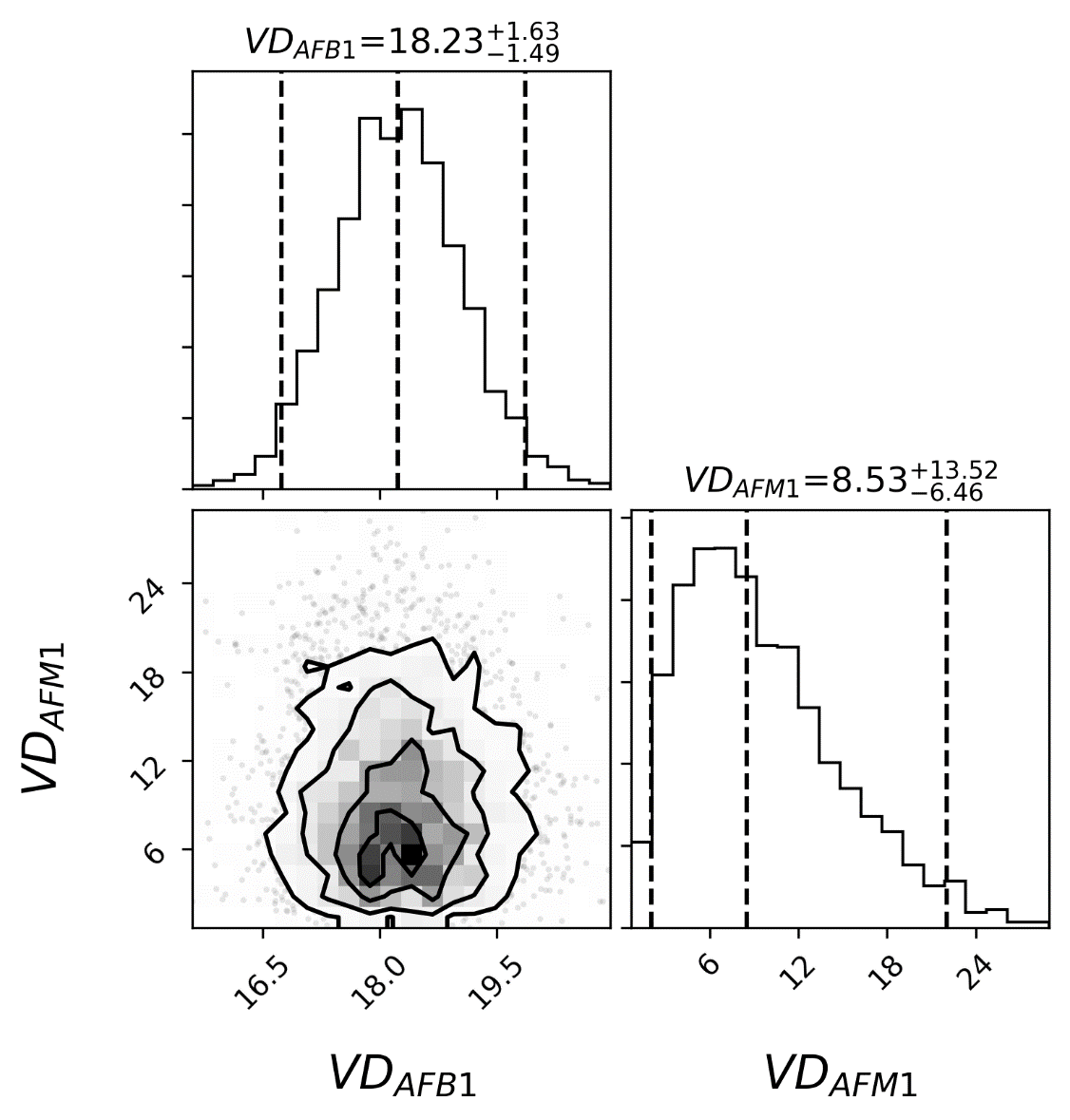


Figure S3: Corner plots of the distribution of the volume of distribution in the study by Guo et al. (Guo, Fan et al. 2021) The dashed lines show the [0.025, 0.5, 0.975] quantiles and the title depicts $X_{-Z}^{+Y}$, where $X$ is the median and Y,Z the distance of the median to the upper, lower quantiles resp

ectively.

## Feeding scenarios

b)

a)


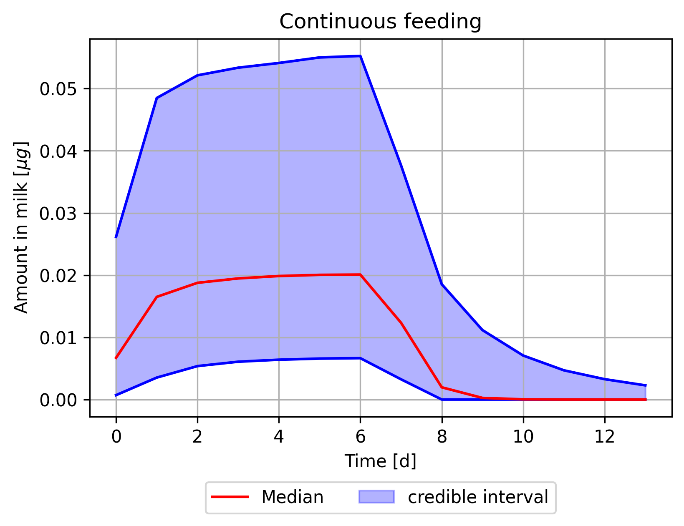

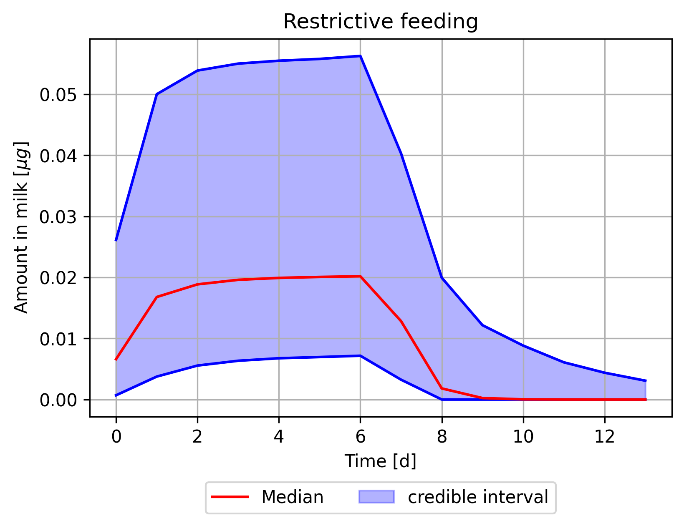


Figure S4: Concentration time profiles of AFM1 in milk in two different scenarios of 1 $\mu g/d$ exposure to AFB1 for 7 days and milking (30 L/d) each day 20:00. a.) Continuous exposure of the animals. b) Strict feeding times at 6:00 and 18:00 each day.

Figure S4 compares continuous exposure throughout the day with a scenario in which the animals have strict feeding times. Only minor differences in the resulting amount of AFM1 in the milk can be observed.

# References

Britzi, M., et al. (2013). "Carry-over of aflatoxin B1 to aflatoxin M1 in high yielding Israeli cows in mid-and late-lactation." Toxins **5**(1): 173-183.

Chopra, R., et al. (1999). "Carryover of Aflatoxin Mt in Milk of Cows Fed Aflatoxin B1 contaminated ration." Indian Journal of Animal Nutrition **16**(2): 103-106.

Frobish, R., et al. (1986). "Aflatoxin residues in milk of dairy cows after ingestion of naturally contaminated grain." Journal of food protection **49**(10): 781-785.

Galvano, F., et al. (1996). "Reduction of carryover of aflatoxin from cow feed to milk by addition of activated carbons." Journal of food protection **59**(5): 551-554.

Giovati, L., et al. (2014). "Vaccination of heifers with anaflatoxin improves the reduction of aflatoxin B1 carry over in milk of lactating dairy cows." PLoS One **9**(4): e94440.

Guo, W., et al. (2021). "In vivo kinetics and biotransformation of aflatoxin B1 in dairy cows based on the establishment of a reliable UHPLC-MS/MS method." Frontiers in Chemistry **9**: 809480.

Jiang, Y., et al. (2018). "Effect of adding clay with or without a Saccharomyces cerevisiae fermentation product on the health and performance of lactating dairy cows challenged with dietary aflatoxin B1." Journal of dairy science **101**(4): 3008-3020.

Kutz, R., et al. (2009). "Efficacy of Solis, NovasilPlus, and MTB-100 to reduce aflatoxin M1 levels in milk of early to mid lactation dairy cows fed aflatoxin B1." Journal of dairy science **92**(8): 3959-3963.

Lamp, J., et al. (2026). "Maize inoculation with aflatoxigenic and biocontrol fungi - toxin transfer from feed into milk and yoghurt." Mycotoxin research **42**.

Maki, C., et al. (2016). "Effects of calcium montmorillonite clay and aflatoxin exposure on dry matter intake, milk production, and milk composition." Journal of dairy science **99**(2): 1039-1046.

Masoero, F., et al. (2007). "Carryover of aflatoxin from feed to milk in dairy cows with low or high somatic cell counts." Animal **1**(9): 1344-1350.

Mood, A. M. (1950). "Introduction to the Theory of Statistics."

Pietri, A., et al. (2009). "Aflatoxin transfer from naturally contaminated feed to milk of dairy cows and the efficacy of a mycotoxin deactivating product." International Journal of Dairy Science **4**(2): 34-42.

Price, R. L., et al. (1985). "Aflatoxin conversion by dairy cattle consuming naturally-contaminated whole cottonseed." Journal of food protection **48**(1): 11-16.

Queiroz, O., et al. (2012). "Effect of adding a mycotoxin-sequestering agent on milk aflatoxin M1 concentration and the performance and immune response of dairy cattle fed an aflatoxin B1-contaminated diet." Journal of dairy science **95**(10): 5901-5908.

Rodrigues, R., et al. (2019). "Feed additives containing sequestrant clay minerals and inactivated yeast reduce aflatoxin excretion in milk of dairy cows." Journal of dairy science **102**(7): 6614-6623.

Rojo, F., et al. (2014). "Comparison of methods to evaluate aflatoxin B1 exposure in dairy cattle and the effect of mycotoxin adsorbents to reduce AFM1 residues in milk." Revista mexicana de ciencias pecuarias **5**(1): 1-15.

Veldman, A., et al. (1992). "Carry-over of aflatoxin from cows' food to milk." Animal Science **55**(2): 163-168.

Walte, H., et al. (2022). "Re-evaluation of aflatoxin M." Journal of Animal and Feed Sciences **31**(4):

Xiong, J., et al. (2015). "Transfer of dietary aflatoxin B1 to milk aflatoxin M1 and effect of inclusion of adsorbent in the diet of dairy cows." Journal of dairy science **98**(4): 2545-2554.
